# Role of CD38 in mediating the effect of Bacillus on acute pancreatitis: a study of mediated Mendelian randomization

**DOI:** 10.3389/fimmu.2024.1452743

**Published:** 2024-11-15

**Authors:** Junhao Xiao, Zhuoyan Tao, Mingjian Luo, Yong Yan, Shaobiao Ke, Benliang Mao, Jiulin Zhan, Zhe Wang, Bailin Wang, Zhiwei Li

**Affiliations:** ^1^ Department of Hepatobiliary Surgery, Dongguan Kanghua Hospital, Dongguan, China; ^2^ Department of Breast Surgery, Shenzhen Futian Maternal and Child Health Center, Shenzhen, China; ^3^ Department of General Surgery, Guangzhou Red Cross Hospital, Guangzhou, China; ^4^ Department of Medical Cosmetology, Shenzhen Guangming District People’s Hospital, Shenzhen, China

**Keywords:** intestinal flora, immune cell traits, acute pancreatitis, Mendelian randomization, mediation analysis

## Abstract

**Background:**

Some studies suggest a potential link between intestinal flora and acute pancreatitis (AP). However, the causal relationships between specific intestinal flora and AP, and the possible mediating role of immune cell traits, remain unclear.

**Methods:**

A genome-wide association study (GWAS) involving 5,959 participants was conducted to identify genetic instrumental variables associated with 473 intestinal flora taxa. Summary statistics for AP were obtained from the UK Biobank. Immune cell traits were also identified using large-scale GWAS summary data. We employed a two-sample bidirectional Mendelian randomization (MR) approach to investigate the causal relationships between intestinal flora, immune cell traits, and AP, with inverse variance weighting (IVW) as the primary statistical method. Sensitivity analyses, including the MR-Egger intercept test, Cochran’s Q test, MR-PRESSO test, and leave-one-out test, were conducted to assess the robustness of our findings. Additionally, we explored whether immune cell traits mediate the pathway from intestinal flora to AP.

**Results:**

11 positive and 11 negative causal relationships were identified between genetic susceptibility in intestinal flora and AP. Furthermore, 19 positive and 9 negative causal relationships were observed between immune cell traits and AP. Notably, CD38 mediated the causal relationship between Bacillus C and AP.

**Conclusions:**

This study is the first to uncover novel causal relationships between various intestinal flora and acute pancreatitis, emphasizing the mediating role of immune cell traits in the pathway from intestinal flora to AP. It also provides new evidence supporting the conditional pathogenicity of the Bacillus genus.

## 1 Introduction

Acute pancreatitis (AP) is a common gastrointestinal disorder characterized by inflammation of the pancreas, which can vary from mild and self-limiting to severe and life-threatening (1, 2). While traditional causes such as gallstones, hyperlipidemia, and alcohol consumption are well-documented, there is growing recognition of the role of intestinal bacterial translocation in the onset of pancreatic infections (3, 4). This emerging understanding highlights the need to consider bacterial translocation as a significant factor in the pathogenesis of AP.

The intestinal flora, comprising approximately 100 trillion microorganisms, plays an important role in regulating host immunity and metabolism, with dysbiosis of the intestinal flora being associated with various diseases, including inflammatory bowel disease and cardiovascular conditions (5, 6). In addition, present research has demonstrated the significant role of the intestinal flora in the pathogenesis of AP, particularly in influencing disease severity. This interaction between the intestinal flora and the pancreas is referred to as the gut-pancreas axis, and alterations in the intestinal flora can impact pancreatic function by modulating gut immunity, intestinal barrier integrity, and metabolite production (7). Conversely, inflammation and abnormal enzyme secretion in the pancreas can disrupt the balance of the intestinal flora, leading to dysregulation of the gut-pancreas axis and exacerbating pancreatic disease (8). In addition, animal studies have demonstrated that the intestinal flora can influence immune responses through metabolites such as short-chain fatty acids (SCFAs) and bile acids (9, 10). In severe AP cases, reduced SCFA levels are associated with disease progression (11). Moreover, the intestinal flora may amplify the host’s inflammatory response by affecting immune cells such as macrophages and T cells (12). Bacteria may enter the pancreas via the lymphatic system, either independently or facilitated by immune cells, establishing a niche within the pancreas (13, 14). This process, potentially driven by immune cell dysfunction, plays an essential role in the pathogenesis of AP.

Mendelian randomization (MR) is a method utilizing genetic variation as an instrumental variable (IV) to evaluate the causal effects of exposures on health outcomes (15, 16). Since genetic variation is established at conception, MR inherently controls for environmental confounders and reduces the risk of reverse causality (15). Two-sample MR, which utilizes independent samples, further minimizes overfitting and sample selection bias, thereby improving the robustness of causal inferences (17). Additionally, Mediation Mendelian Randomization (MMR) enables the investigation of mediating effects between genetic variation and outcomes, providing insights into underlying biological mechanisms (18).

Although MR-related investigations have indicated a potential causal relationship between certain intestinal flora components and AP (19), further research is needed to elucidate these relationships, particularly given the complexity of the microbiota and the genetic diversity across different populations. Such investigations could offer a solid scientific basis for precision medicine approaches in the management of AP.

In this study, we hypothesize that the intestinal flora and immune cells collectively influence the development of AP, with immune cells potentially serving as mediators between the microbiota and AP. We performed MR analysis to explore the causal relationships among the intestinal flora, immune cells and AP, and assessed the mediating role of immune cells in these relationships. Additionally, we investigated whether genetic susceptibility to AP affects the intestinal flora and immune cells through reverse causality analysis.

## 2 Materials and methods

### 2.1 Study design

**Figure 1:**
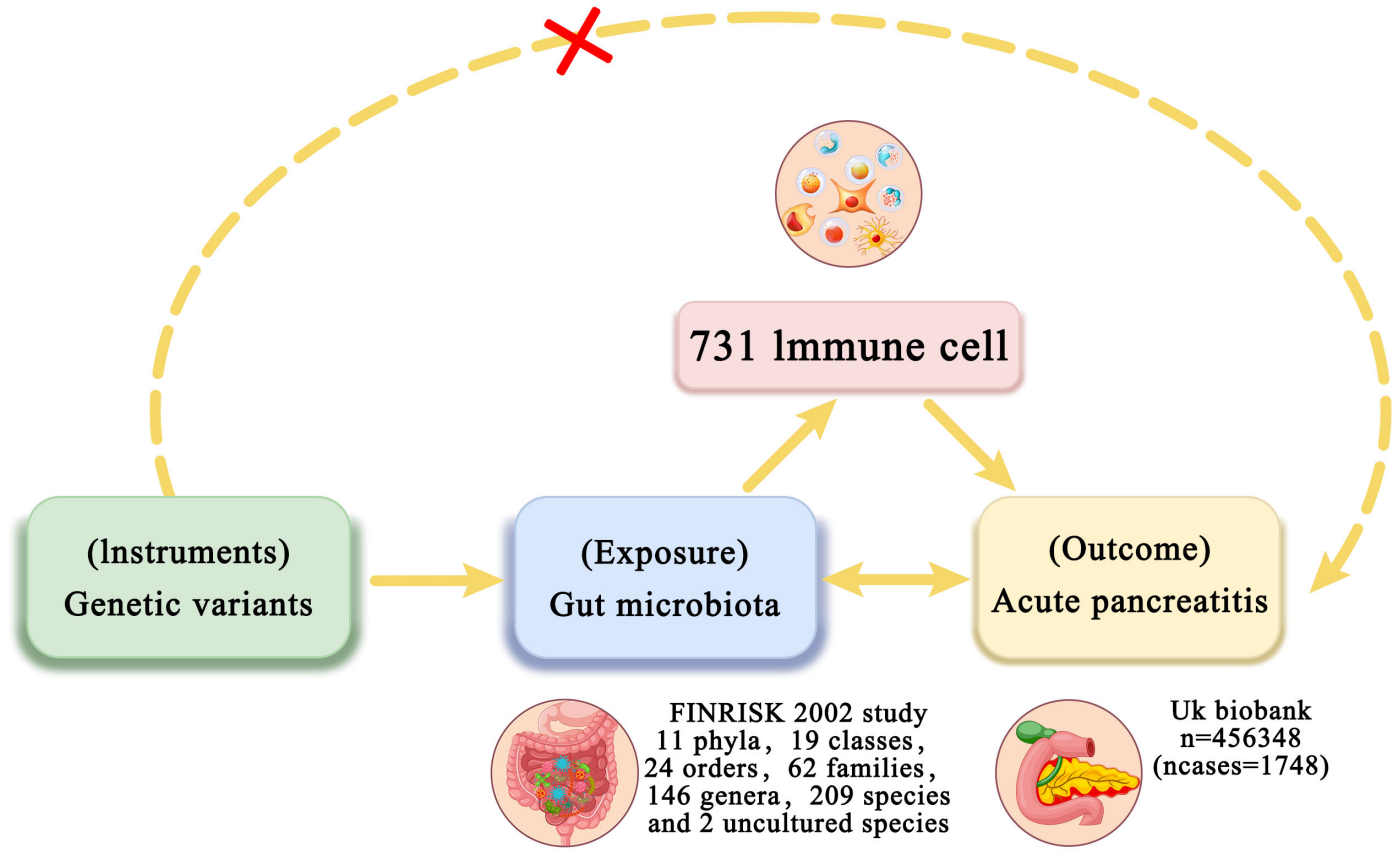
Overview of bidirectional and mediation MR analysis. First, MR analysis was used to investigate the causal relationship between intestinal flora and AP. Subsequently, a reverse MR analysis was performed on the identified intestinal flora to establish unidirectional causal relationships. Second, 731 immune cell traits (mediators) were selected for subsequent mediation analysis. Finally, a two-step MR analysis was conducted to detect potential mediating metabolites.

This study performed two-sample MR analysis using genome-wide association study (GWAS) summary data to investigate the genetic associations between intestinal flora and AP. Two-step and multivariable Mendelian randomization (MVMR) methods were then performed to examine the mediating role of immune cell traits in the relationship between intestinal flora and AP. In the first step, two-sample MR was utilized to assess the causal effects of intestinal flora and immune cell traits on AP and to identify traits with significant causal associations. The second step involved evaluating whether the identified intestinal flora could influence AP through immune cell traits and calculating the mediation effect (**Figure 1**). We also utilized publicly available summary statistics for intestinal flora, immune cell traits, and AP from previously published studies or consortia while adhering to the STROBE-MR reporting guidelines (20). As the data used were GWAS summary-level data, all informed consent and ethical approvals were obtained and reported in the original studies.

### 2.2 Data source

The intestinal flora data were derived from fecal metagenomic sequencing conducted by Qin et al. The study population comprised 5,959 participants from the FINRISK 2002 study, which included men and women aged 25-74 from six geographical regions in Finland (21). The microbiome data included 473 gut microbial taxa, encompassing 11 phyla, 19 classes, 24 orders, 62 families, 146 genera, 209 species, and 2 uncultured species (UBA 3855 sp900316885 and CAG-81 sp000435795). The study also considered potential confounding factors such as diet and antibiotic use to ensure reproducibility across cohorts. Complete summary statistics for microbial taxa with genome-wide significant associations are available in the NHGRI-EBI GWAS Catalog (22) (https://www.ebi.ac.uk/gwas/), accession numbers GCST90032172 to GCST90032644. Data on 731 immune cell traits (accession numbers Ebi-a-GCST 0001391 to Ebi-a-GCST 0002121) were obtained from the GWAS Catalog (Genome-Wide Association Studies, https://gwas.mrcieu.ac.uk/). These traits are categorized into six groups: B cells, conventional dendritic cells (CDC), mature T cells, monocytes, myeloid cells, T cells, B cells, natural killer cells (TBNK), and regulatory T cells (Treg). The immune cell traits included absolute cell (AC) counts (n=118), relative cell (RC) counts (n=192), median fluorescence intensity (MFI) reflecting surface antigen levels (n=389), and morphological parameters (MP) (n=32). To avoid potential sample overlap, outcome data directly from Finland were excluded. Therefore, GWAS summary statistics for AP were retrieved from the UK Biobank, which included 1,748 cases and 454,600 controls of European ancestry (23) (https://www.ebi.ac.uk/gwas/).

### 2.3 Instrumental variable selection

In this study, single nucleotide polymorphisms (SNPs) were used as IVs to explore the causal relationship between exposure and outcome at the genetic level in MR analyses. Initially, SNPs significantly associated with intestinal flora were selected using the standard GWAS threshold (*P* < 5 × 10^-5^). However, this approach yielded a limited number of IVs, which could compromise data reliability. To address this, we adopted a more inclusive screening threshold (*P* < 1 × 10^-5^), as recommended by previous MR studies (19, 24). To ensure the independence of the selected SNPs, linkage disequilibrium (LD) clumping was applied to exclude SNPs with high correlation (r² < 0.001, kb = 10,000). SNPs with palindromic or ambiguous characteristics were systematically excluded from the analysis. The strength of each SNP as an IV was assessed using the F-statistic (F = beta²/SE²). An F-statistic greater than 10 was considered adequate to prevent weak instrument bias in two-sample MR models (25). The same criteria were applied to the selection of IVs for immune cell traits.

### 2.4 Statistical analyses

Two-sample MR analyses were conducted to evaluate the causal relationships between intestinal flora, immune cells, and AP. For traits with a single IV, the Wald ratio test estimated the association between the IV and AP. For traits with multiple IVs, five commonly used multiple regression analysis techniques were employed: inverse variance weighted (IVW) analysis (26), weighted median estimation (WME) (27), MR-Egger regression (28), MR-PRESSO (29), and the weighted model (30). IVW was used as the primary method due to its robustness in the absence of horizontal pleiotropy. Quadratic analyses were also performed using WME and MR-Egger regression methods. The results were expressed as odds ratios (OR) with 95% confidence intervals (CI). Statistical significance was defined as a p-value for IVW less than 0.05, with IVW results consistent with WME and MR-Egger results. Bonferroni-corrected p-values were applied for each level of intestinal flora characterization (phylum, order, family, and genus), defined as *P* < 0.05/n, where n represents the effective number of independent bacterial taxa at the corresponding taxonomic level. For immune cell traits, a Bonferroni-corrected p-value of 6.8 × 10^-5^ (0.05/731) was used. A p-value less than 0.05 but above the Bonferroni-corrected threshold was considered suggestive of correlation. Cochran’s Q test was employed to assess heterogeneity among SNPs, with a Q-statistical significance of *P* < 0.05 indicating potential heterogeneity (31). Leave-one-out analyses were conducted to evaluate the influence of individual SNPs on the results by excluding each SNP and performing an IVW approach on the remaining SNPs. MR-PRESSO and MR-Egger regressions were used to detect and correct for potential horizontal pleiotropy, with *P* < 0.05 considered significant for horizontal pleiotropy, indicating unreliable causality. MR-PRESSO was specifically used to identify significant outliers and correct for horizontal pleiotropic effects by removing these outliers. All statistical analyses were performed using R version 4.3.2, with MR analysis conducted using the R-based software packages “TwoSampleMR” (32) and “MR_PRESSO” (29).

### 2.5 Reverse MR analysis

To investigate whether AP has a causal effect on the identified intestinal flora and immune cell traits, we conducted a reverse MR analysis. In this analysis, SNPs associated with AP were used as IVs, with AP serving as the exposure and the significant intestinal flora and immune cell traits as outcomes. SNPs significantly associated with AP (*P* < 5 × 10^-5^) were selected as IVs, with linkage disequilibrium excluded by clumping at r² < 0.001, kb = 10,000.

### 2.6 Mediation analysis

Moreover, we included intestinal flora and immune cell traits with unidirectional causal effects on AP in the mediation analysis through bidirectional MR analysis and assessed the role of the intestinal flora in immune cell characterization and, if so, performed multifactorial MR analysis to determine if immune cells mediate the relationship between intestinal flora and AP. The total effect of intestinal flora on AP was calculated, and this total effect was decomposed into a direct effect and a mediated effect through immune cell traits. The effect of intestinal flora on immune cells (β1) and the effect of immune cell traits on AP (β2) were determined. The mediated effect was calculated as β1 × β2, while the direct effect was obtained by subtracting the mediated effect from the total effect. Consistency between the indirect and total effects was assessed, and the mediation percentage was calculated by dividing the mediated effect by the total effect.

## 3 Results

### 3.1 Instrumental variable selection

In this study, we performed instrumental variable selection on GWAS data for 473 intestinal flora and 731 immune cell traits. At the *P* < 1 × 10^-5^ level, all intestinal flora and immune cell traits had eligible SNPs. These 9,238 SNPs were selected as IVs for intestinal flora, as detailed in **Supplementary Table S1**. Additionally, 18,728 SNPs were selected as IVs for immune cell traits, as listed in **Supplementary Table S2**.

### 3.2 Causal relationship between intestinal flora and acute pancreatitis

**Figure 2:**
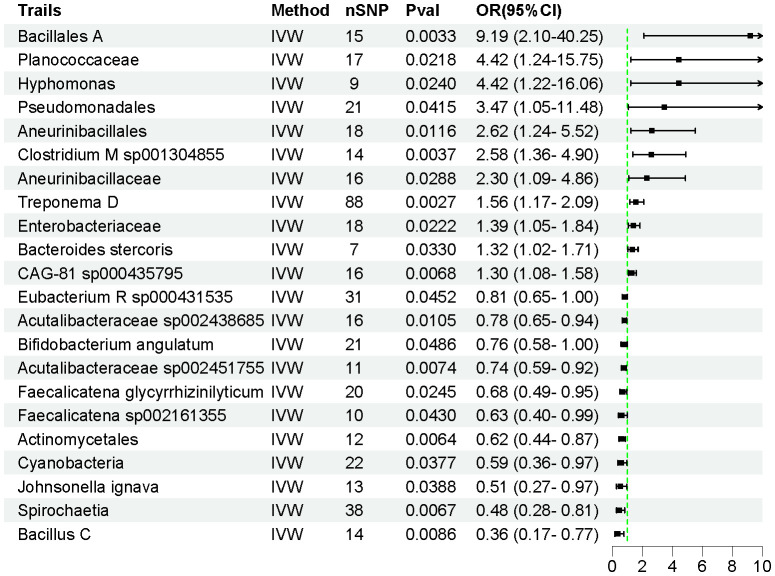
The forest plot illustrates the causal relationships between intestinal flora and AP.

Using the IVW method, we identified 22 intestinal flora taxa that may be causally associated with AP. As shown in **Figure 2**, 11 intestinal flora were genetically predicted to be associated with an increased risk of AP. Specifically, Bacillales A (OR = 9.188, 95% CI = 2.097–40.25, *P* = 0.003), Planococcaceae (OR = 4.423, 95% CI = 1.242–15.75, *P* = 0.022), Hyphomonas (OR = 4.419, 95% CI = 1.216–16.06, *P* = 0.024), Pseudomonadales (OR = 3.471, 95% CI = 1.049–11.48, *P* = 0.041), Aneurinibacillales (OR = 2.616, 95% CI = 1.240–5.519, *P* = 0.012), Clostridium M sp001304855 (OR = 2.582, 95% CI = 1.362–4.896, *P* = 0.004), Aneurinibacillaceae (OR = 2.303, 95% CI = 1.090–4.864, *P* = 0.029), Treponema D (OR = 1.560, 95% CI = 1.166–2.086, *P* = 0.003), Enterobacteriaceae (OR = 1.390, 95% CI = 1.048–1.843, *P* = 0.022), Bacteroides stercoris (OR = 1.323, 95% CI = 1.023–1.712, *P* = 0.033), and uncultured CAG-81 sp000435795 (OR = 1.304, 95% CI = 1.076–1.580, *P* = 0.006) were associated with an increased risk of AP development.

Conversely, Eubacterium R sp000431535 (OR = 0.807, 95% CI = 0.654–0.995, P = 0.045), Acutalibacteraceae sp002438685 (OR = 0.781, 95% CI = 0.647–0.944, *P* = 0.011), Bifidobacterium angulatum (OR = 0.760, 95% CI = 0.579–0.998, P = 0.048), Acutalibacteraceae sp002451755 (OR = 0.737, 95% CI = 0.590–0.927, *P* = 0.007), Faecalicatena glycyrrhizinilyticum (OR = 0.680, 95% CI = 0.486–0.952, *P* = 0.025), Faecalicatena sp002161355 (OR = 0.627, 95% CI = 0.399–0.985, *P* = 0.043), Actinomycetales (OR = 0.618, 95% CI = 0.438–0.873, *P* = 0.006), Cyanobacteria (OR = 0.588, 95% CI = 0.357–0.970, *P* = 0.038), Johnsonella ignava (OR = 0.513, 95% CI = 0.273–0.966, *P* = 0.039), Spirochaetia (OR = 0.477, 95% CI = 0.279–0.815, *P* = 0.006), and Bacillus C (OR = 0.358, 95% CI = 0.167–0.771, *P* = 0.009) were associated with a reduced risk of AP (**Supplementary Table S3**). The F-statistics for all instrumental variables were greater than 10, indicating no weak instrumental bias in our analysis. Detailed information on 450 SNPs across the 22 intestinal flora taxa is provided in **Supplementary Table S4**.

### 3.3 Causal relationship between immune cell traits and acute pancreatitis

**Figure 3:**
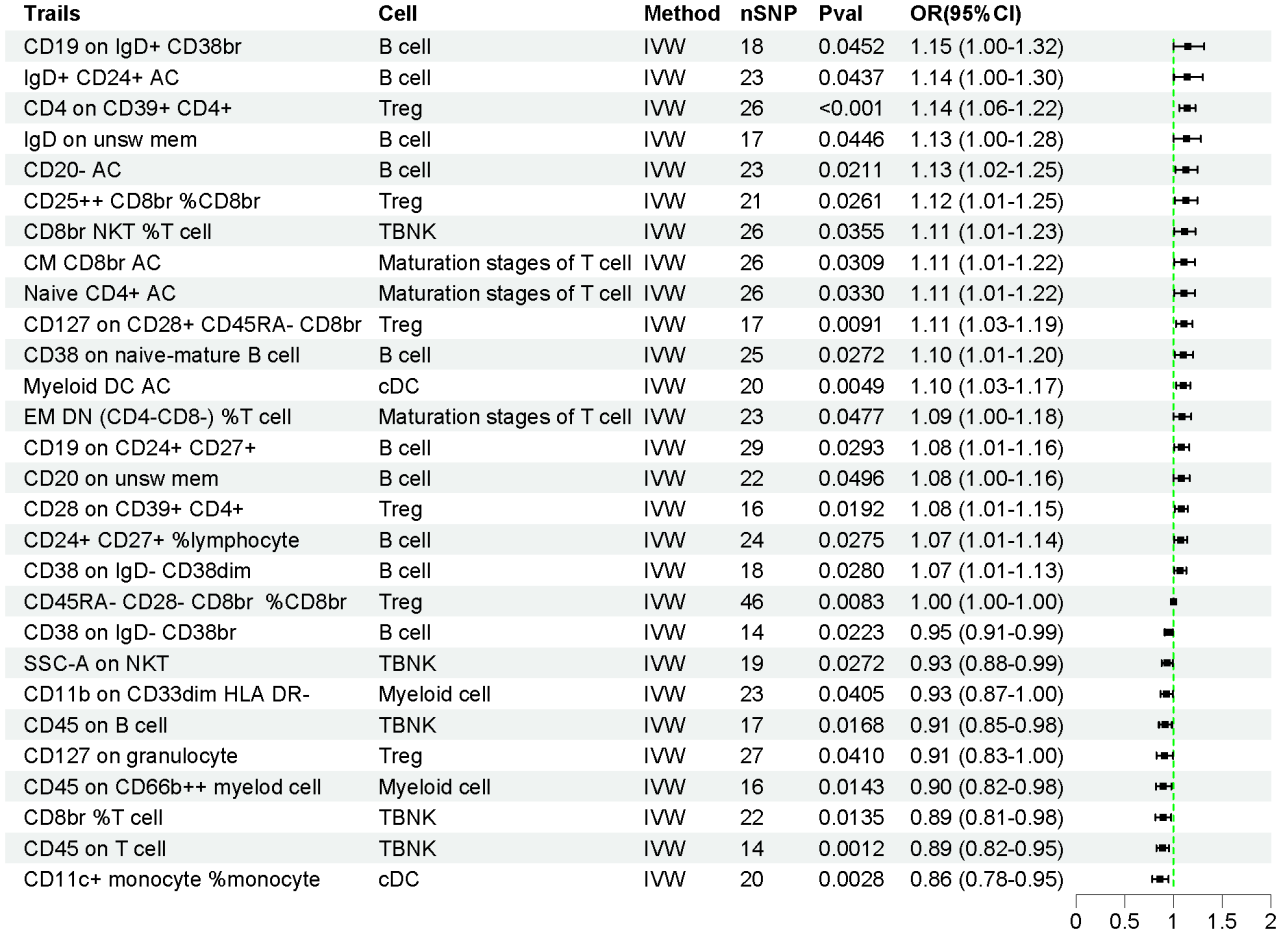
The forest plot illustrates the causal relationships between immune cell traits and AP.

The effects of 731 immune cell traits on AP were assessed using the IVW method. As shown in **Figure 3**, the genetic prediction of 28 immune cell traits was found to be causally associated with AP. Of these, 19 immune cell traits were significantly associated with an increased risk of AP, while 9 immune cell traits were significantly associated with a decreased risk of AP (**Supplementary Table S5**). Detailed information on 628 SNPs across the 28 immune cell traits is provided in **Supplementary Table S6**.

### 3.4 Sensitivity analysis

Using the MR-Egger regression intercept method, no genetic pleiotropy was detected for the intestinal flora, and MR-PRESSO analysis confirmed the absence of horizontal pleiotropy in the MR study between intestinal flora and AP (*P* > 0.05). Cochran’s Q test showed no significant heterogeneity (*P* > 0.05, **Supplementary Table S7**). The leave-one-out sensitivity test indicated that the causal relationship between intestinal flora and AP was not influenced by the omission of any single SNP, demonstrating the robustness of the MR analysis (**Supplementary Figures S1**-**4**). Scatter plots illustrated the overall effect of intestinal flora on AP (**Supplementary Figures S5**-**8**). Additionally, fixed-effect IVW analysis provided causal estimates and 95% CIs for each SNP (**Supplementary Figures S9**-**12**).

Similarly, the MR-Egger regression intercept method did not detect genetic pleiotropy for immune cell traits, and MR-PRESSO analysis confirmed no horizontal pleiotropy in the MR study (*P* > 0.05). Cochran’s Q test showed no significant heterogeneity *(P* > 0.05, **Supplementary Table S8**). The leave-one-out sensitivity test (**Supplementary Figures S13**-**17**), scatter plots (**Supplementary Figures S18**-**22**), and fixed-effect IVW analysis (**Supplementary Figures S23**-**27**) were also employed for immune cell traits, and the results were similarly reliable.

### 3.5 Reverse Mendelian randomization analysis

To verify the directionality of causality, we performed a reverse MR analysis. This approach allowed us to assess the causal effect of disease outcomes on exposure, thereby reducing the potential interference of reverse causality. With this method, we could more accurately determine the direction of causality and enhance the credibility of the results from the forward MR analysis. In the reverse MR analysis, we used AP as the exposure data and screened instrumental variables based on a threshold of *P* < 5 × 10^-5^. A total of 80 SNPs were included in the analysis as IVs after exclusion of chain imbalance. The results showed that there was no reverse causality between AP and any of the 20 intestinal flora. Notably, AP was also causally associated with Hyphomonas (OR = 1.008, 95% CI = 1.002–1.014, *P* = 0.013) and Pseudomonadales (OR = 1.005, 95% CI = 1.002–1.008, *P* = 0.002). Thus, we established a unidirectional causal relationship between AP and the 20 intestinal flora (**Supplementary Table S9**). Similarly, reverse MR analysis between AP and 28 immune cell traits showed that there was an inverse causal relationship between AP and CD28 on CD39+ CD4+ (OR = 1.044, 95% CI = 1.006–1.082, *P* = 0.007) and CD45 on CD66b++ myeloid cells (OR = 1.051 95% CI = 1.014~1.090, *P* = 0.013). Therefore, we established a unidirectional causal relationship between AP and 26 immune cell traits (**Supplementary Table S10**).

### 3.6 Mediation analysis

In this study, both intestinal flora and immune cell traits were found to be causally involved in AP. Therefore, we hypothesized that immune cell characteristics mediate the relationship between intestinal flora and AP. We further conducted mediation analyses for intestinal flora and immune cell traits where unidirectional causality existed. The results demonstrated that CD38 on naive-mature B cells mediated the causal relationship between Bacillus C and AP (OR = 1.965, 95% CI = 1.205–3.206, *P* = 0.006). In the pathway where Bacillus C influences acute pancreatitis (AP), Bacillus C has a protective causal relationship with AP (**Table 1**). CD38 on naive-mature B cells also has an independent causal relationship with AP and is a risk factor for AP (**Table 2**). Under the mediating effect of CD38 on naive-mature B cells, Bacillus C increases the risk of AP (**Table 3**). We calculated the total effect of Bacillus C on AP to be -1.026. The effect of Bacillus C on CD38 on naive-mature B cells is β1 = 0.676, and the effect of CD38 on naive-mature B cells on AP is β2 = 0.097. The indirect effect is 0.066, and the direct effect is -1.092. Therefore, we conclude that although Bacillus C can reduce the risk of AP, it exhibits pathogenicity under the mediation of CD38 on naive-mature B cells. These findings provide new evidence supporting Bacillus as a conditionally pathogenic bacterium.

**Table 1:** The causal relationship between Bacillus C and AP was evaluated using five methods.

Exposure	Outcome	Method	OR	95%CI	*P*
Bacillus C	AP	MR Egger	0.315	0.054~1.841	0.224
		Weighted median	0.287	0.103~0.797	0.017
		IVW	0.358	0.167~0.771	0.009
		Simple mode	0.300	0.053~1.709	0.198
		Weighted mode	0.245	0.053~1.131	0.095

**Table 2:** The causal relationship between CD38 on naive-mature B cell and AP was evaluated using five methods.

Exposure	Outcome	Method	OR	95%CI	*P*
CD38	AP	MR Egger	1.166	1.022~1.330	0.032
		Weighted median	1.075	0.934~1.238	0.315
		IVW	1.102	1.011~1.201	0.027
		Simple mode	1.229	0.994~1.519	0.068
		Weighted mode	1.127	0.980~1.296	0.108

**Table 3:** The mediating role of CD38 on naive-mature B cell in the pathway between Bacillus C and AP was evaluated using five methods.

Exposure	Outcome	Mediator	Method	OR	95%CI	*P*
Bacillus C	AP	CD38	MR Egger	2.363	0.822~6.789	0.136
			Weighted median	2.105	1.052~4.212	0.035
			IVW	1.965	1.205~3.206	0.006
			Simple mode	1.230	0.411~3.679	0.718
			Weighted mode	2.298	0.872~6.057	0.116

## 4 Discussion

AP is a prevalent pancreatic disorder that imposes a significant health and economic burden on society (33). Emerging research suggests a potential causal relationship between the intestinal flora and AP (34). Despite this, advancements in therapies, risk identification, and survival outcomes for AP have been relatively stagnant compared to other gastrointestinal diseases (35). Therefore, it is essential to conduct further research to identify risk factors associated with AP and to enhance human health and clinical management of this condition.

In this study, we employed MR to investigate the potential causal relationship between the intestinal flora and AP. We analyzed the abundance of 473 common intestinal flora species and their association with AP, and further explored the possible mediating role of immune cell traits in this relationship. To our knowledge, no previous studies have reported on the causal relationship between this specific set of intestinal flora and AP. Our findings could contribute to identifying intestinal flora with potential impacts on AP, offering new insights into its pathogenesis and informing future research.

Our findings revealed that specific intestinal flora can function as both risk and protective factors for AP. We identified that a genetic predisposition to 11 intestinal flora species was associated with an increased risk of AP, whereas another 11 species were linked to a decreased risk. Furthermore, 19 immune cell traits were found to significantly elevate the risk of developing AP, while 9 traits were associated with a reduced risk. Reverse MR analysis confirmed unidirectional causal relationships between 21 intestinal flora species and 26 immune cell traits with AP. The diversity of the intestinal flora is pivotal for maintaining human health. Compared to healthy individuals, patients with AP show elevated levels of Bacteroidetes and Proteobacteria, and reduced levels of Firmicutes and Actinobacteria (36). Specifically, members of the Firmicutes phylum, including Eubacterium, Acutalibacteraceae, Faecalicatena and Bacillus, as well as members of the Actinobacteria phylum, such as Bifidobacterium and Actinomycetales, are associated with a reduced risk of AP. Conversely, members of the Proteobacteria phylum, including Hyphomonas, Pseudomonadales, and Enterobacteriaceae, as well as members of the Bacteroidetes phylum, such as Bacteroides, are linked to an increased risk of AP. Additionally, we observed that Enterobacteriaceae may have a curative effect, while Bifidobacterium spp. appears to offer protective benefits. Bifidobacterium spp., which are known for their positive effects on intestinal health, help to maintain intestinal barrier function and reduce systemic inflammatory responses. These actions contribute to protection against AP. Tan et al. reported a significant increase in potentially pathogenic bacteria, such as Enterobacteriaceae and Enterococcus spp., and a significant decrease in beneficial bacteria, such as Bifidobacterium spp., in patients with moderate and severe acute pancreatitis (MAP and SAP) (37). Moreover, Li et al. demonstrated that Bifidobacterium spp. and its metabolite lactate prevented AP by inhibiting both pancreatic and systemic inflammatory responses (38). Yu et al. identified Eubacterium hallii, a major butyrate-producing bacterium, as significantly reduced in patients with MAP and SAP (39). Our findings align with these observations, showing a protective role of Eubacterium against AP. The genus Bacillus, which includes microorganisms utilized in the production of various fermented foods and as probiotic supplements, is known for its roles in enzyme, antibiotic, and bioactive peptide production (40).

Studies have demonstrated that Bacillus coagulans, as a probiotic, can enhance digestive health, boost immune function, and aid in the treatment of diarrhea and irritable bowel syndrome (IBS) (41). While many strains of the genus Bacillus are generally regarded as harmless or beneficial, they can exhibit pathogenicity under certain conditions and are thus considered conditionally pathogenic. For instance, Bacillus cereus, which is commonly found in soil and food, can multiply and produce toxins when food is improperly stored, resulting in food poisoning (42). Additionally, it can cause severe invasive infections in immunocompromised individuals or those with contaminated medical devices (43). Bacillus pumilus may lead to skin and soft tissue infections or even sepsis, particularly in post-surgical patients or immunosuppressed individuals (44). Bacillus spp. is also believed to have a dual role in AP. Some studies suggest that certain Bacillus spp. might exacerbate pancreatic inflammation through toxin or metabolite production (45). Conversely, other Bacillus strains may offer protective effects due to their anti-inflammatory and immunomodulatory properties (46). Our findings reflect this dual role: Bacillales A is associated with an increased risk of AP, while Bacillus C is linked to a decreased risk. The intestinal flora may influence AP by disrupting the gut barrier, altering metabolite production, and affecting immune cell function. For instance, animal studies have shown that rats with depleted intestinal flora exhibit less pancreatic damage and lower plasma levels of interleukin (IL)-17A, tumor necrosis factor (TNF)-α, and IL-1β compared to rats with intact microbiota (47). Additionally, research by Li et al. indicated that intestinal flora can affect AP severity by interacting with the inflammatory vesicle NLRP3 in a mouse model, suggesting that the intestinal flora may mediate immune responses (48). To further explore this, we conducted a mediation MR analysis. Our results revealed that CD38 on naive-mature B cells mediates the causal relationship between Bacillus C and AP, changing its role from a protective factor to a pathogenic one. CD38, primarily expressed in immune cells in response to cytokines, endotoxins, and interferons, regulates the immune system and induces inflammation through mechanisms such as calcium mobilization, protein phosphorylation, and cytokine release (49, 50). Several studies have established a link between CD38 and inflammatory diseases. For instance, increased infiltration of CD38+ plasma cells has been noted in the synovium of patients with early rheumatoid arthritis (51). Proteomic analyses of intestinal tissues from healthy controls and patients with inflammatory bowel disease have revealed upregulation of CD38, with heightened CD38 protein expression observed in inflamed areas of the colonic mucosa in patients with ulcerative colitis (52). Additionally, research by Orabi et al. has suggested that aberrant intracellular Ca2+ release mediated by CD38 contributes to bile acid-induced pancreatitis and acinar cell injury (53). Therefore, while Bacillus C may possess protective properties, its mediation through CD38 on naive-mature B cells could elevate the risk of AP by influencing immune cell interactions. This finding highlights the significant role of immune cell function in the relationship between the intestinal flora and AP.

The results presented in this study might have some significant clinical implications. First, by elucidating the causal relationship between the intestinal flora and AP, we have identified potential targets for personalized treatment strategies. Future interventions could focus on targeting specific harmful bacterial populations or enhancing the abundance of beneficial microbes to prevent and treat AP. Second, dietary interventions, prebiotics, and probiotics aimed at modulating the intestinal flora may offer potential benefits for improving outcomes in AP. However, a randomized controlled trial has shown that prophylactic administration of probiotics not only failed to reduce the risk of infectious complications in AP patients but was also associated with a 2.5-fold increase in mortality risk (54). Our findings challenge the indiscriminate use of probiotics, underscoring the need for careful selection of appropriate strains and personalized management based on the patient’s immune status to avoid potential adverse effects in clinical practice.

Overall, we identified numerous intestinal flora causally associated with AP through MR analysis, with the findings aligning with previous studies. The reliability of these results is supported by inverse MR and sensitivity analyses. Our research also revealed that different strains within the same phylum or genus can have varying effects on AP. Notably, we identified a previously uncultured strain linked to major depression in prior studies. Through mediator MR analysis, we discovered that CD38 on naive-mature B cells mediates the relationship between Bacillus C and AP, altering its role from protective to pathogenic. These insights underscore the significant role of intestinal flora in driving pancreatitis and suggest that immune cell dysfunction may be a critical factor. However, the specific molecular mechanisms by which the intestinal flora influences AP through immune cell regulation require further investigation, which could pave the way for developing new therapeutic strategies. Personalized intervention strategies based on intestinal flora show great clinical promise but need to be validated through additional clinical trials to assess their safety and efficacy. Furthermore, examining how genetic and environmental factors jointly regulate the intestinal flora and its impact on AP, along with functional research on previously uncultured strains associated with AP, could uncover new disease mechanisms and therapeutic targets and potentially provide a foundation for precision treatment approaches for AP.

Despite providing valuable insights into the causal relationship between intestinal flora and AP, this study had several limitations that should be acknowledged. First, the research was conducted exclusively using a European dataset, which might restrict the generalizability of the findings to other ethnic and geographical groups. Second, the intestinal flora is inherently transient and variable, and we could not assess changes in its relative abundance, introducing potential uncertainties into our genetic predictions. Additionally, MR studies have some inherent limitations, such as the assumption of no pleiotropy and the reliance on well-characterized genetic instruments. Lastly, we used broader thresholds, which may introduce the risk of weak instrument bias, and while we investigated the mediating role of immune cell traits, the specific underlying mechanisms remain unclear and warrant further investigation.

## 5 Conclusion

In this study, we comprehensively explored the causal relationships between intestinal flora, immune cell traits, and acute pancreatitis (AP). We identified 11 positive and 11 negative causal relationships between genetic susceptibility in the intestinal flora and AP. There were 19 positive and 9 negative causal effects between immune cell traits and AP. Additionally, we found a bidirectional causal relationship between the identified intestinal flora and AP, and two bidirectional causal relationships between immune cell traits and AP. CD38 on naive-mature B cells mediated the pathway through which Bacillus C affects AP, increasing the risk of AP under its influence.

## Data Availability

The datasets presented in this study can be found in online repositories. The names of the repository/repositories and accession number(s) can be found in the article/**Supplementary Material**.
