# Supplementary figures and images for: Role of CD38 in mediating the effect of Bacillus on acute pancreatitis: a study of mediated Mendelian randomization
Source: Front Immunol. 2024 Nov 15;15:1452743. doi: 10.3389/fimmu.2024.1452743 (PMC11604618; doi:10.3389/fimmu.2024.1452743)

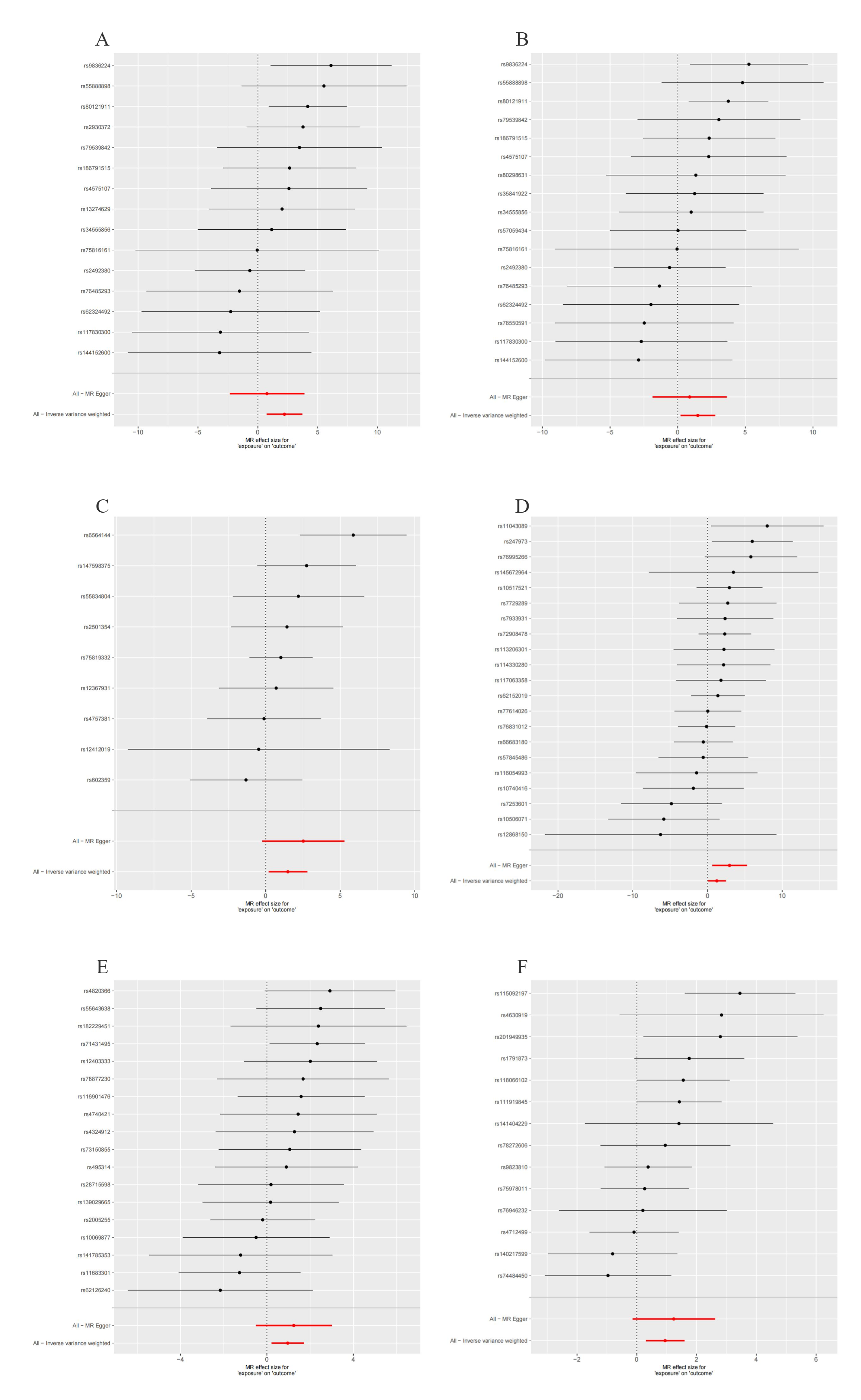

Supplement: Supplementary file 1 [file DataSheet1.zip › supplement figureS1_S12/Figure_S1.tiff]

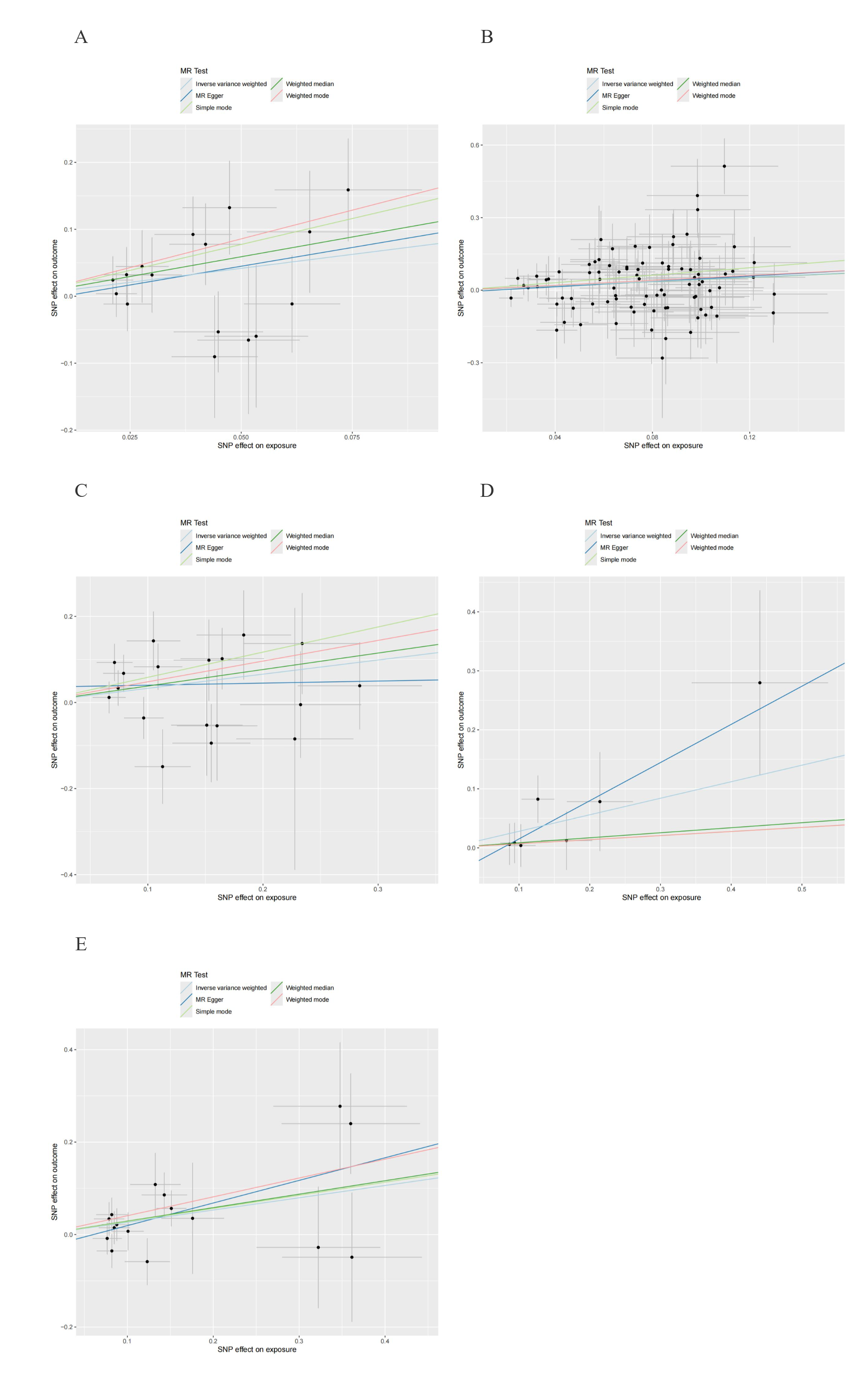

Supplement: Supplementary file 1 [file DataSheet1.zip › supplement figureS1_S12/Figure_S10.tiff]

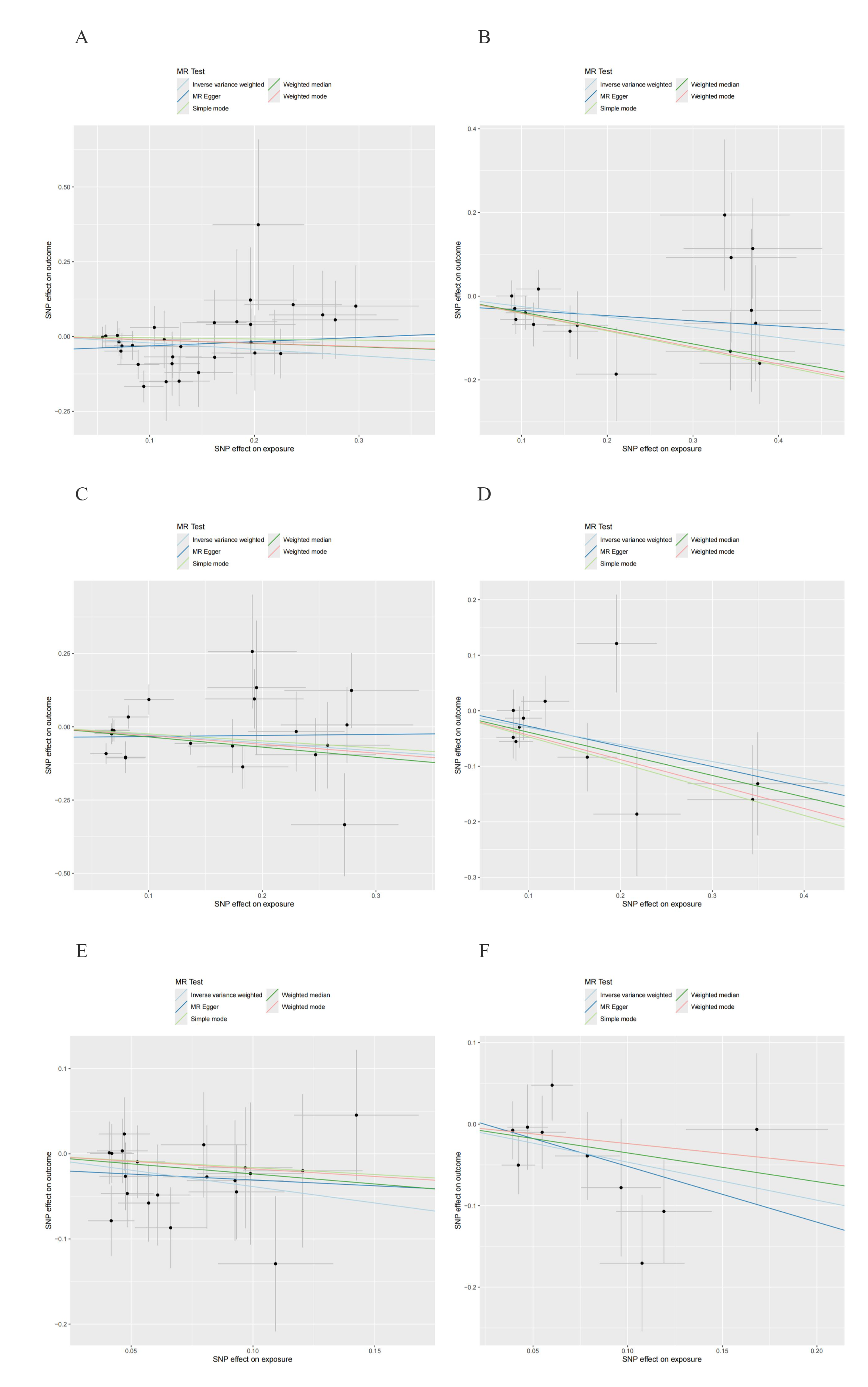

Supplement: Supplementary file 1 [file DataSheet1.zip › supplement figureS1_S12/Figure_S11.tiff]

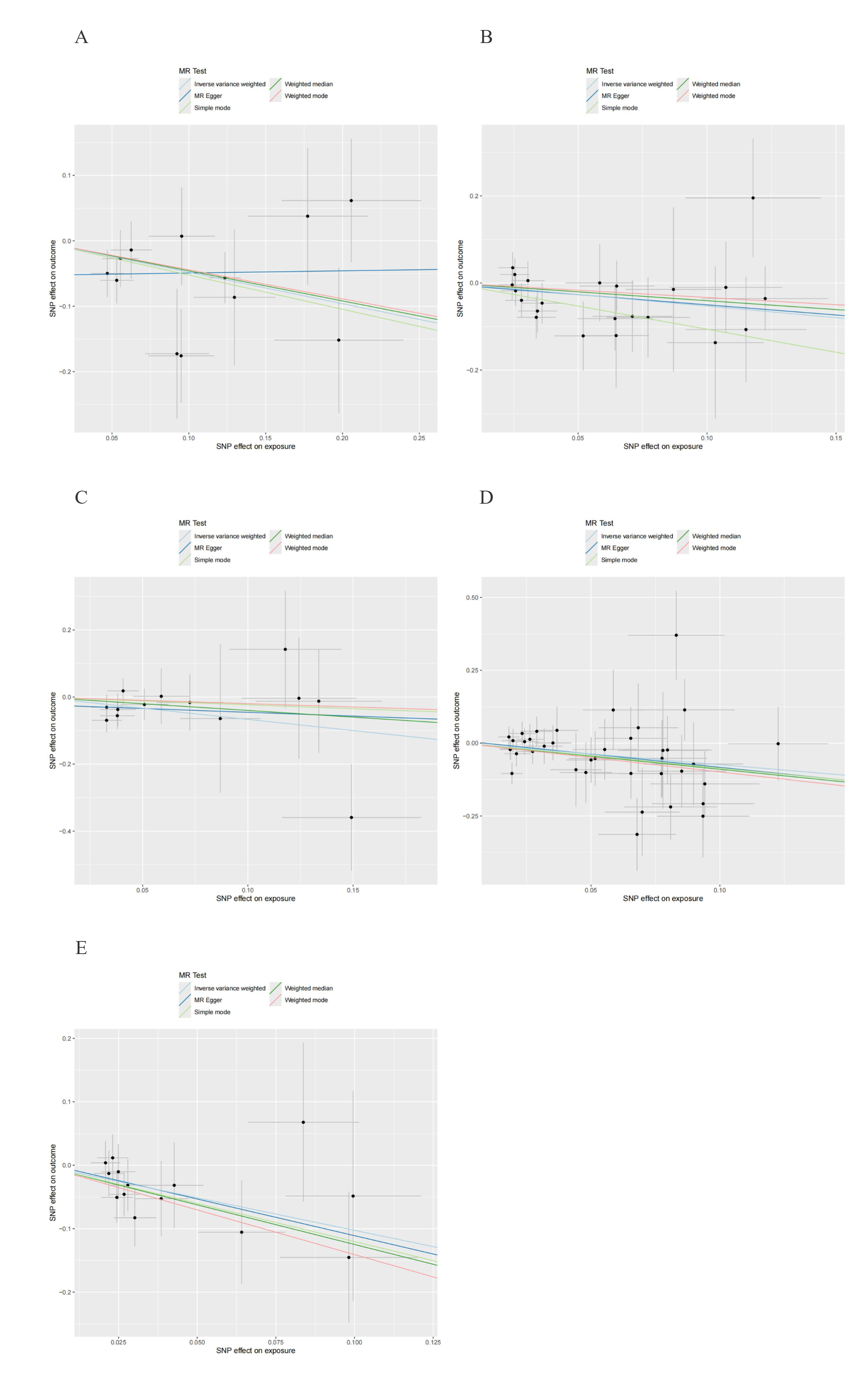

Supplement: Supplementary file 1 [file DataSheet1.zip › supplement figureS1_S12/Figure_S12.tiff]

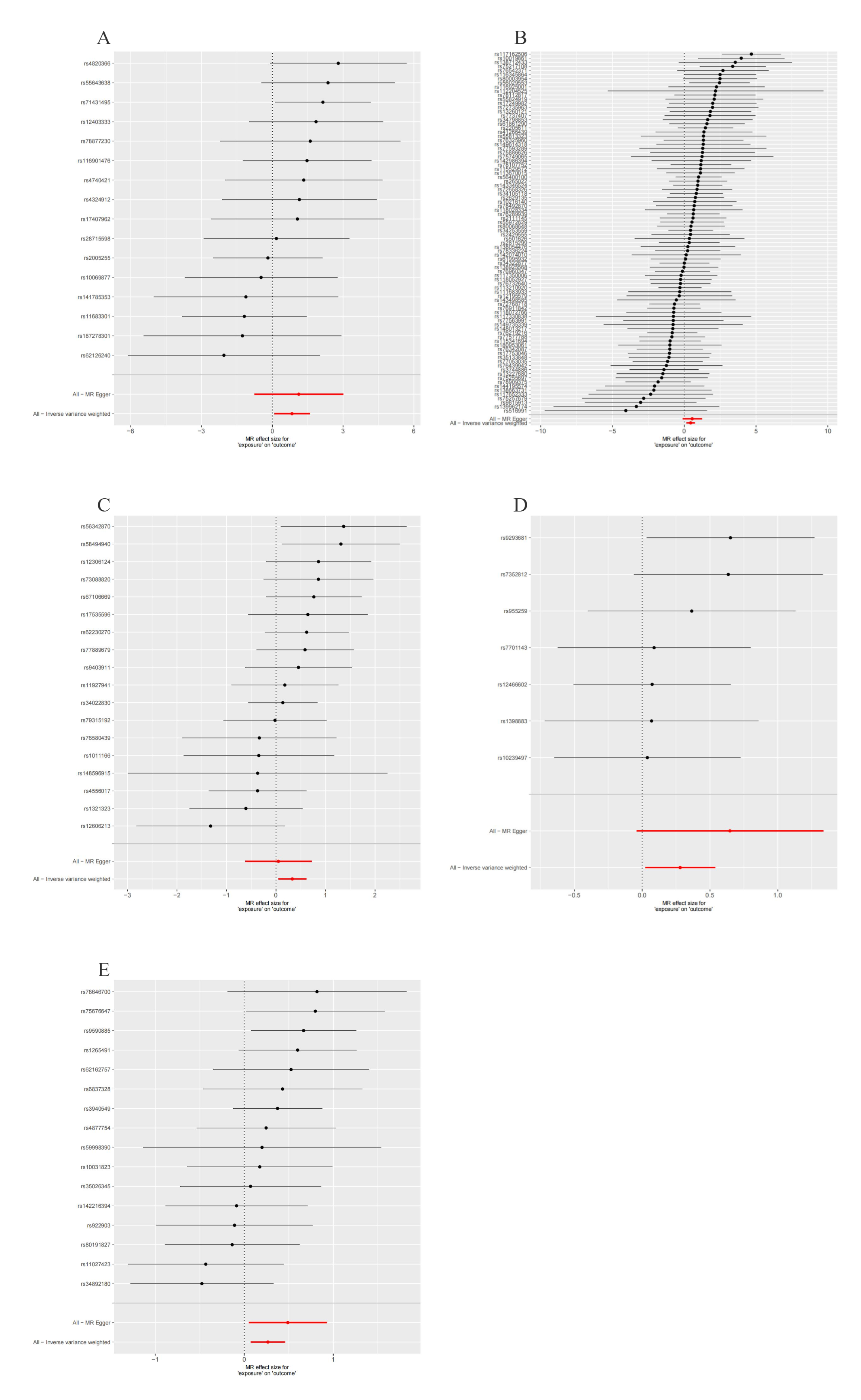

Supplement: Supplementary file 1 [file DataSheet1.zip › supplement figureS1_S12/Figure_S2.tiff]

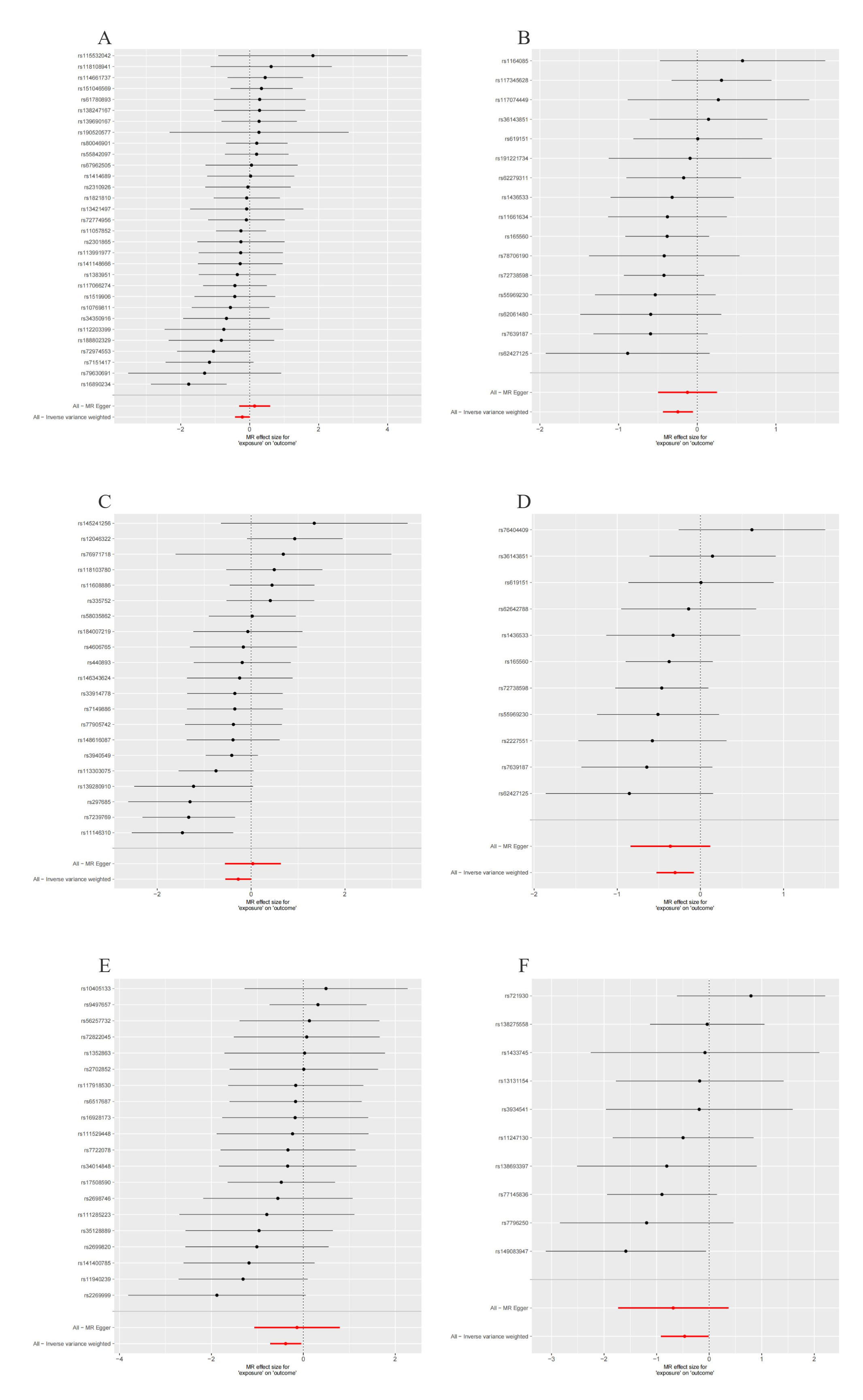

Supplement: Supplementary file 1 [file DataSheet1.zip › supplement figureS1_S12/Figure_S3.tiff]

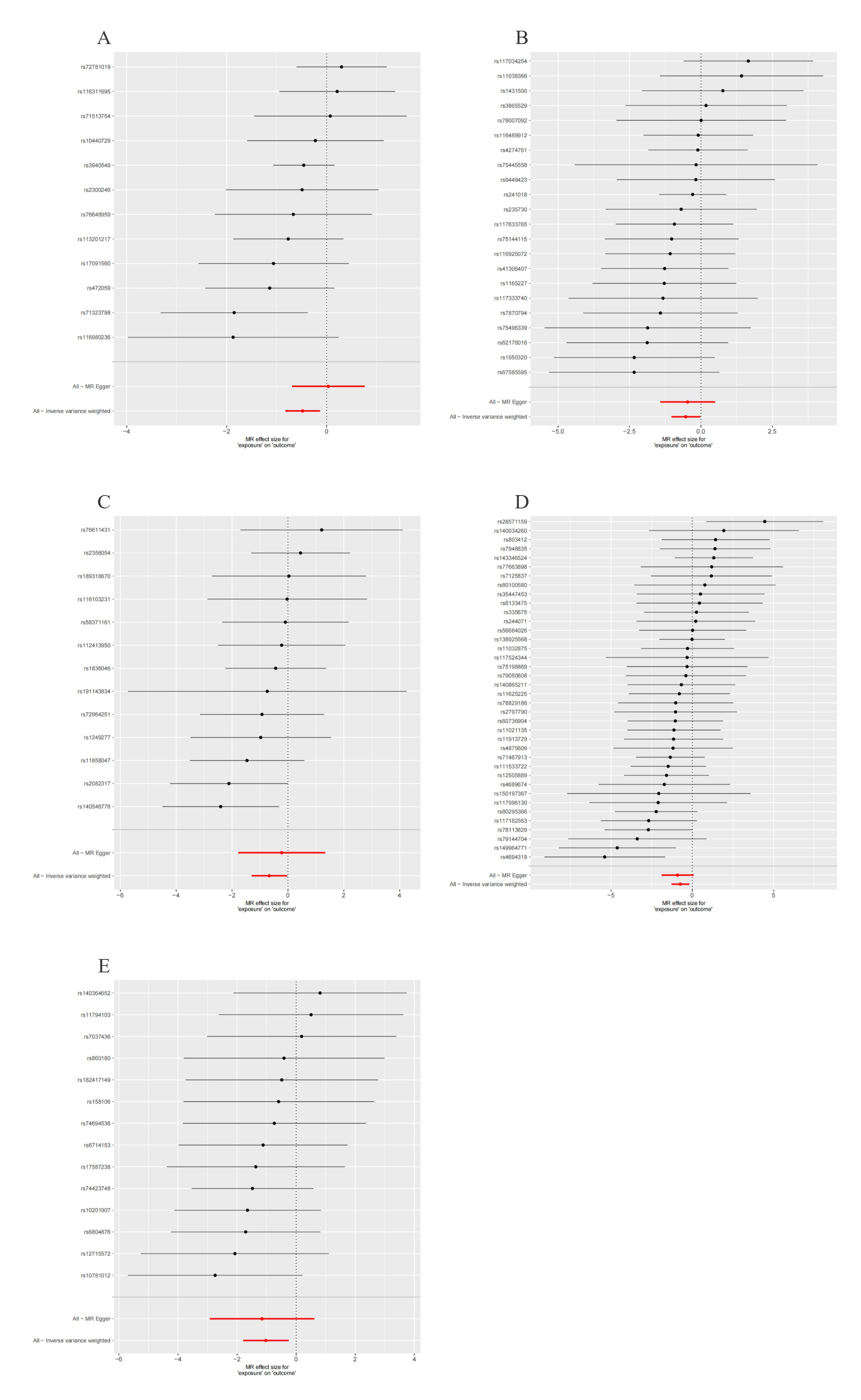

Supplement: Supplementary file 1 [file DataSheet1.zip › supplement figureS1_S12/Figure_S4.tiff]

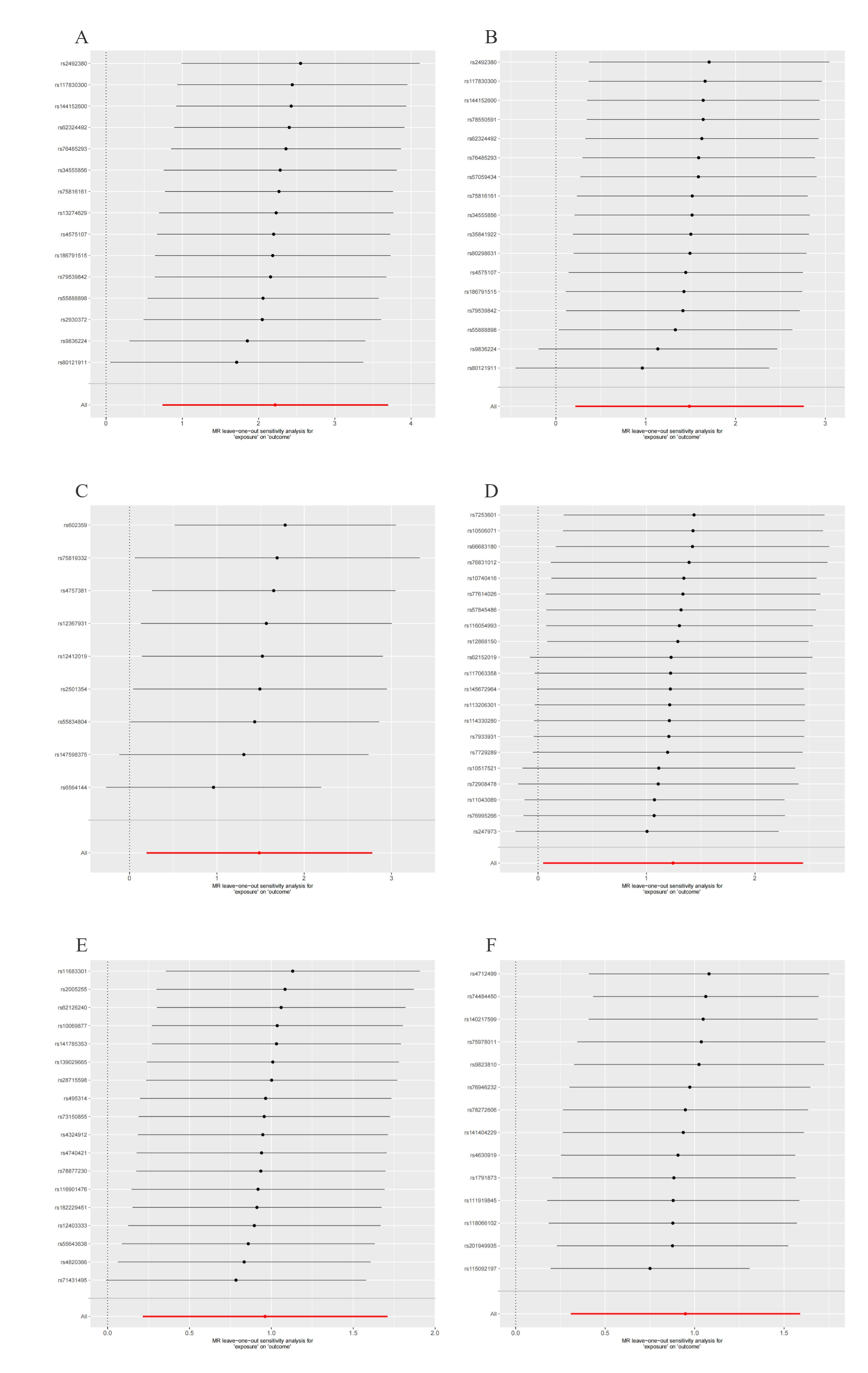

Supplement: Supplementary file 1 [file DataSheet1.zip › supplement figureS1_S12/Figure_S5.tiff]

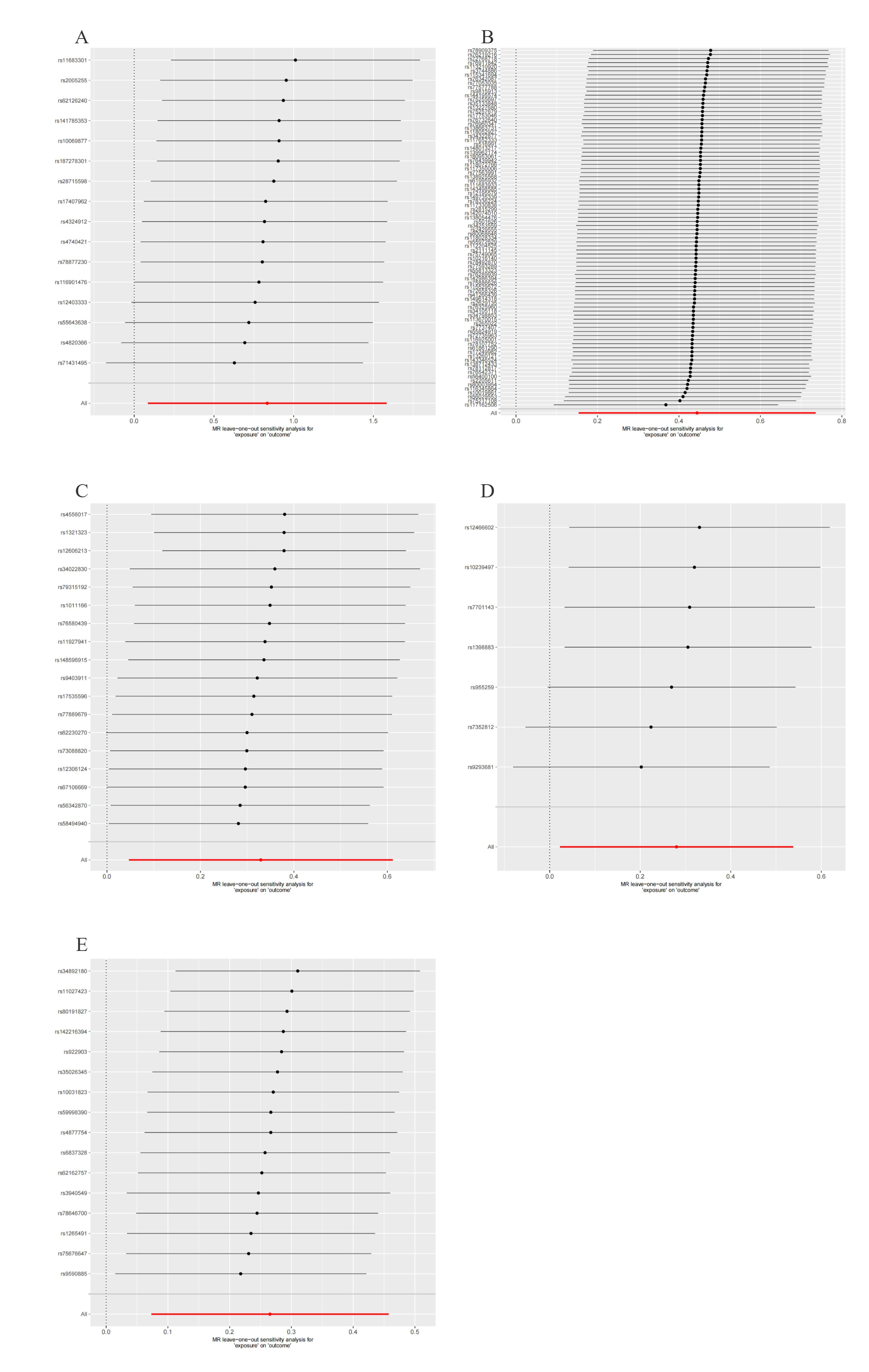

Supplement: Supplementary file 1 [file DataSheet1.zip › supplement figureS1_S12/Figure_S6.tiff]

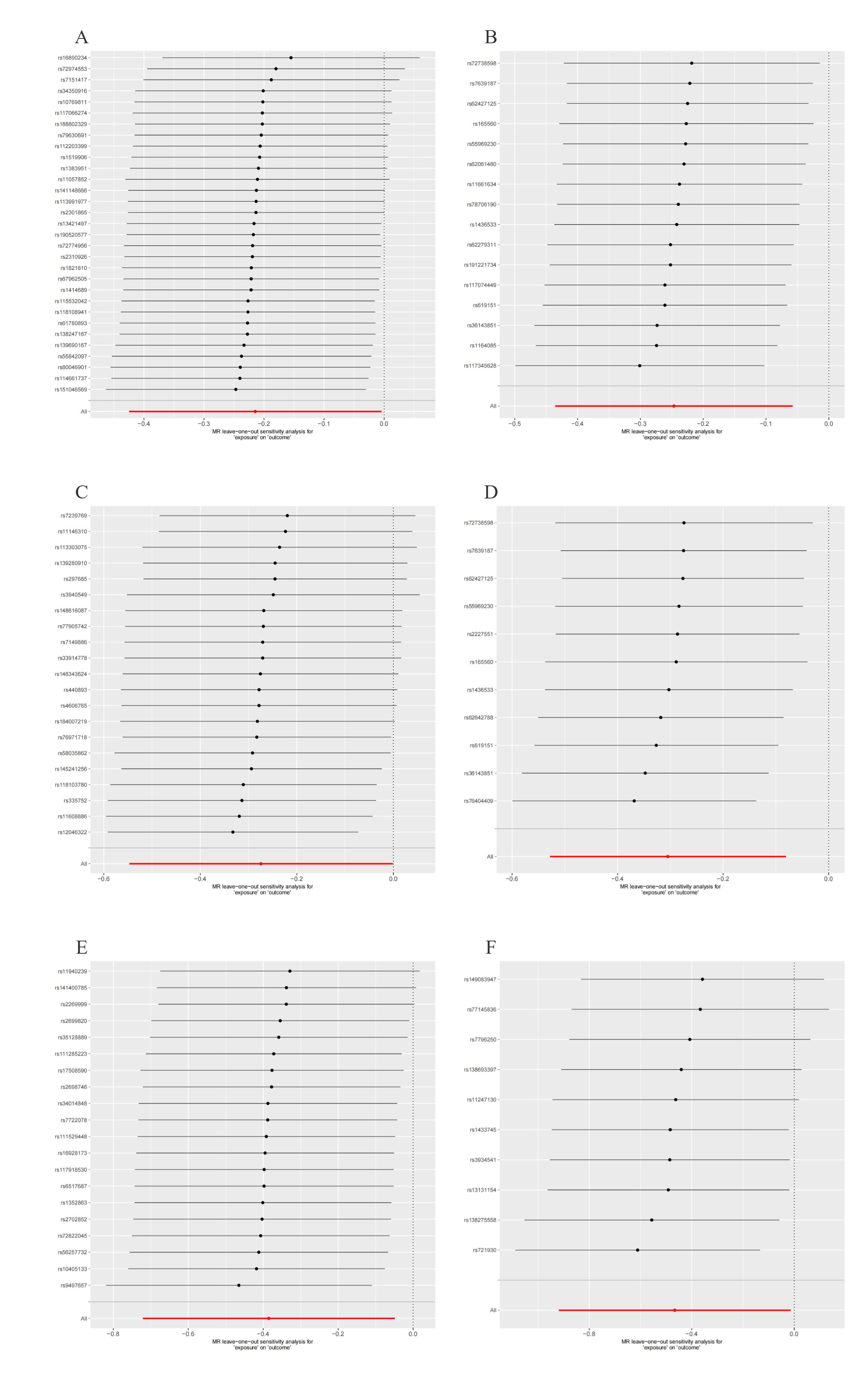

Supplement: Supplementary file 1 [file DataSheet1.zip › supplement figureS1_S12/Figure_S7.tiff]

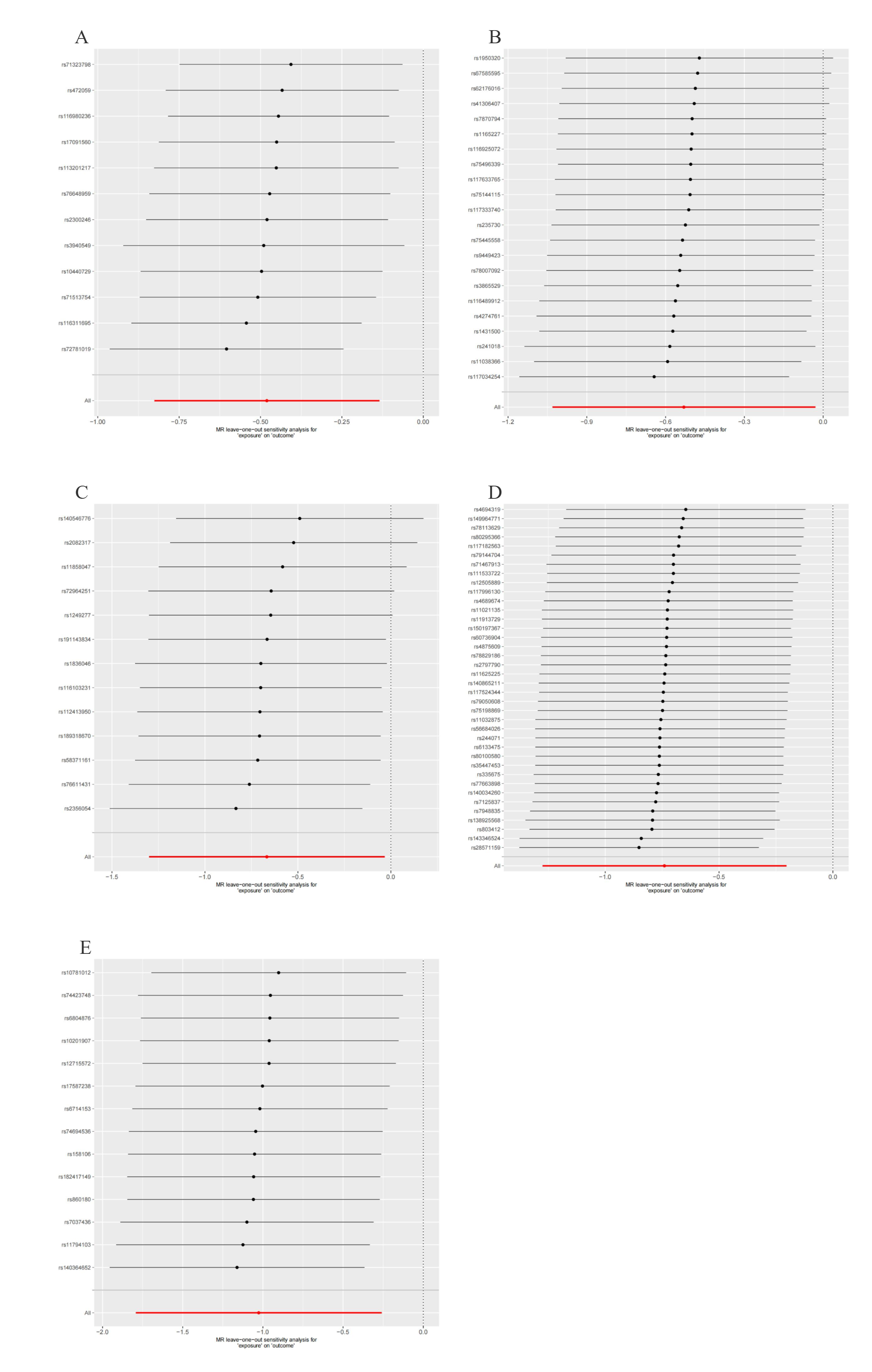

Supplement: Supplementary file 1 [file DataSheet1.zip › supplement figureS1_S12/Figure_S8.tiff]

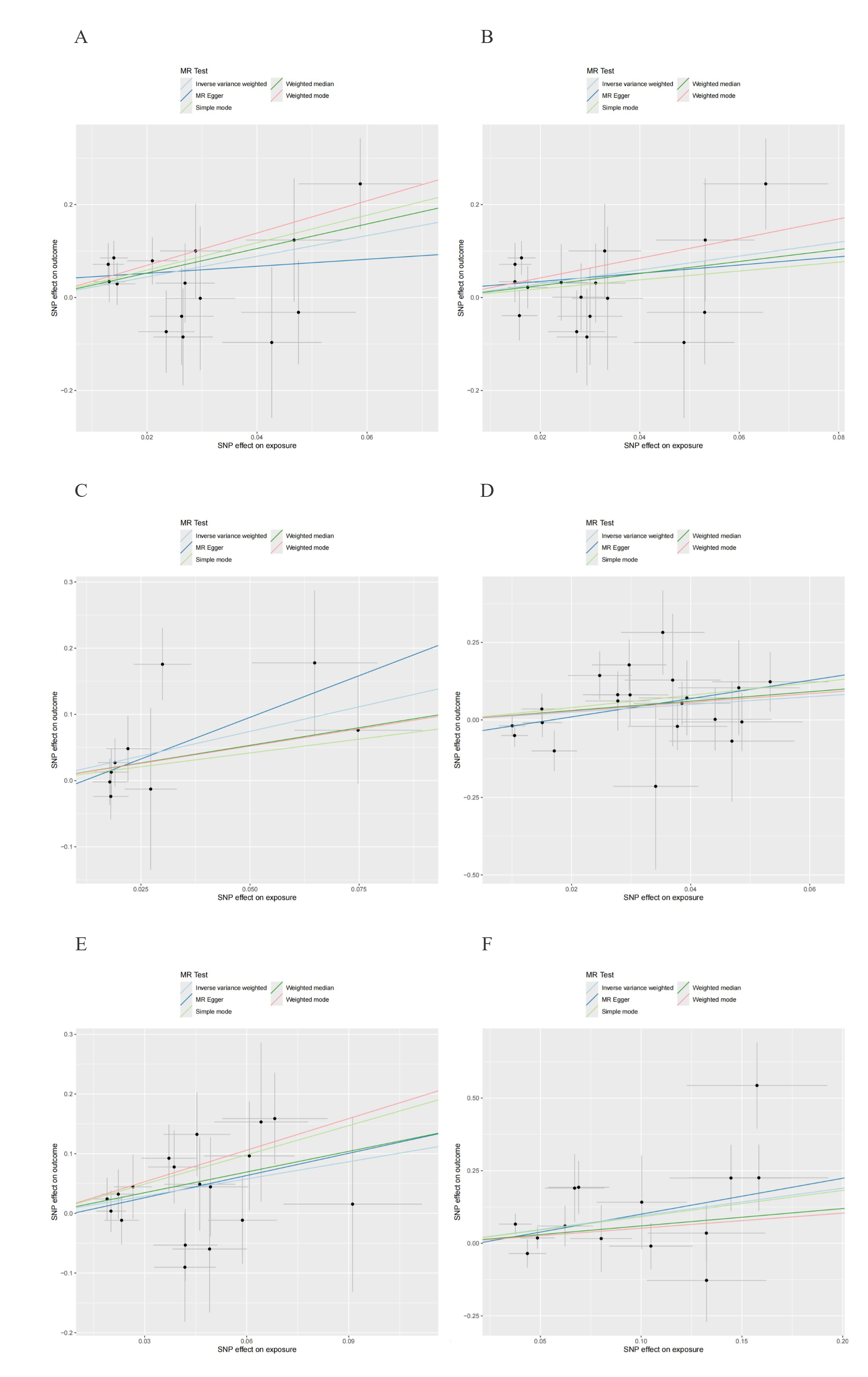

Supplement: Supplementary file 1 [file DataSheet1.zip › supplement figureS1_S12/Figure_S9.tiff]

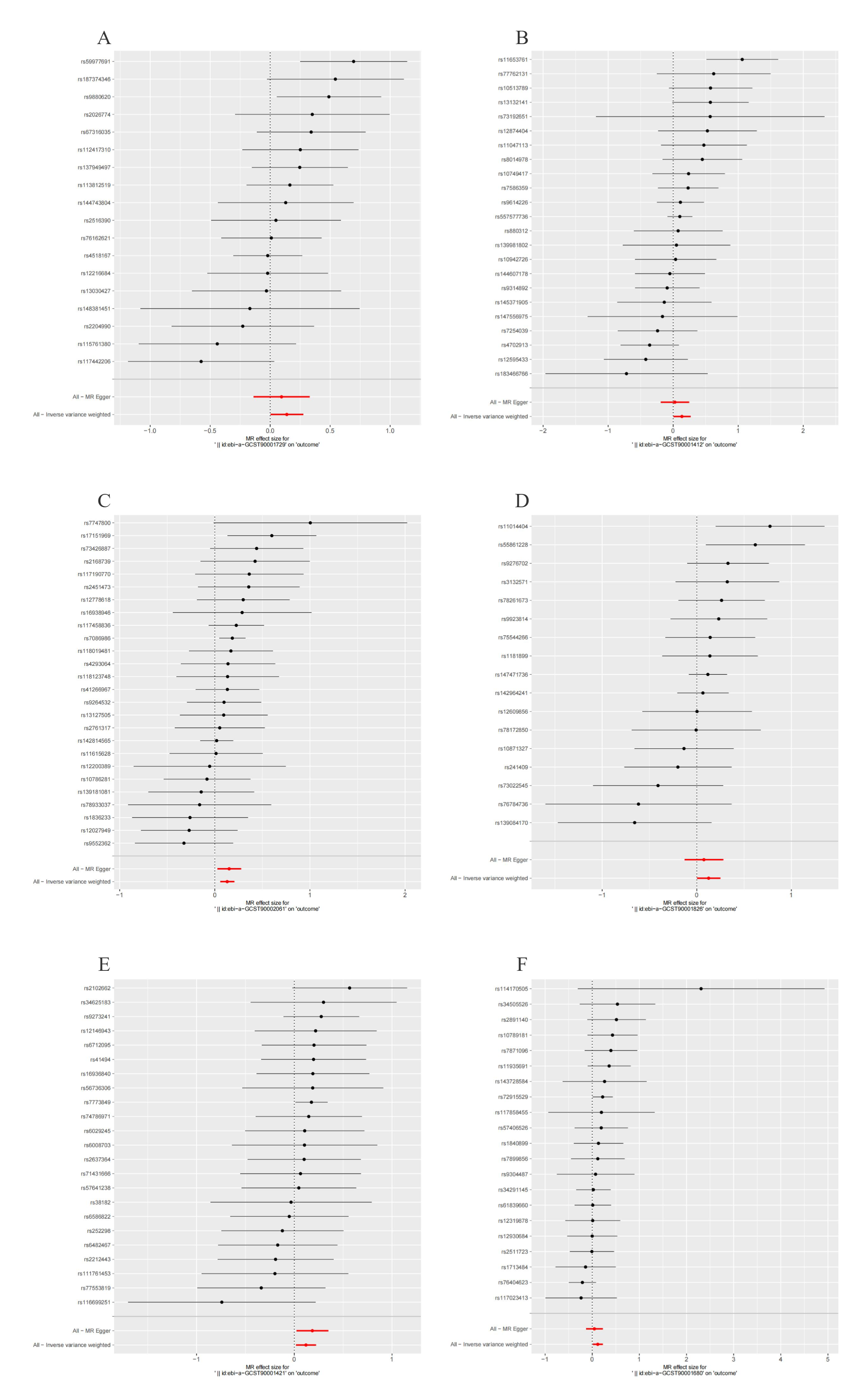

Supplement: Supplementary file 2 [file DataSheet2.zip › Supplement figureS13_S27/Figure_S13.tiff]

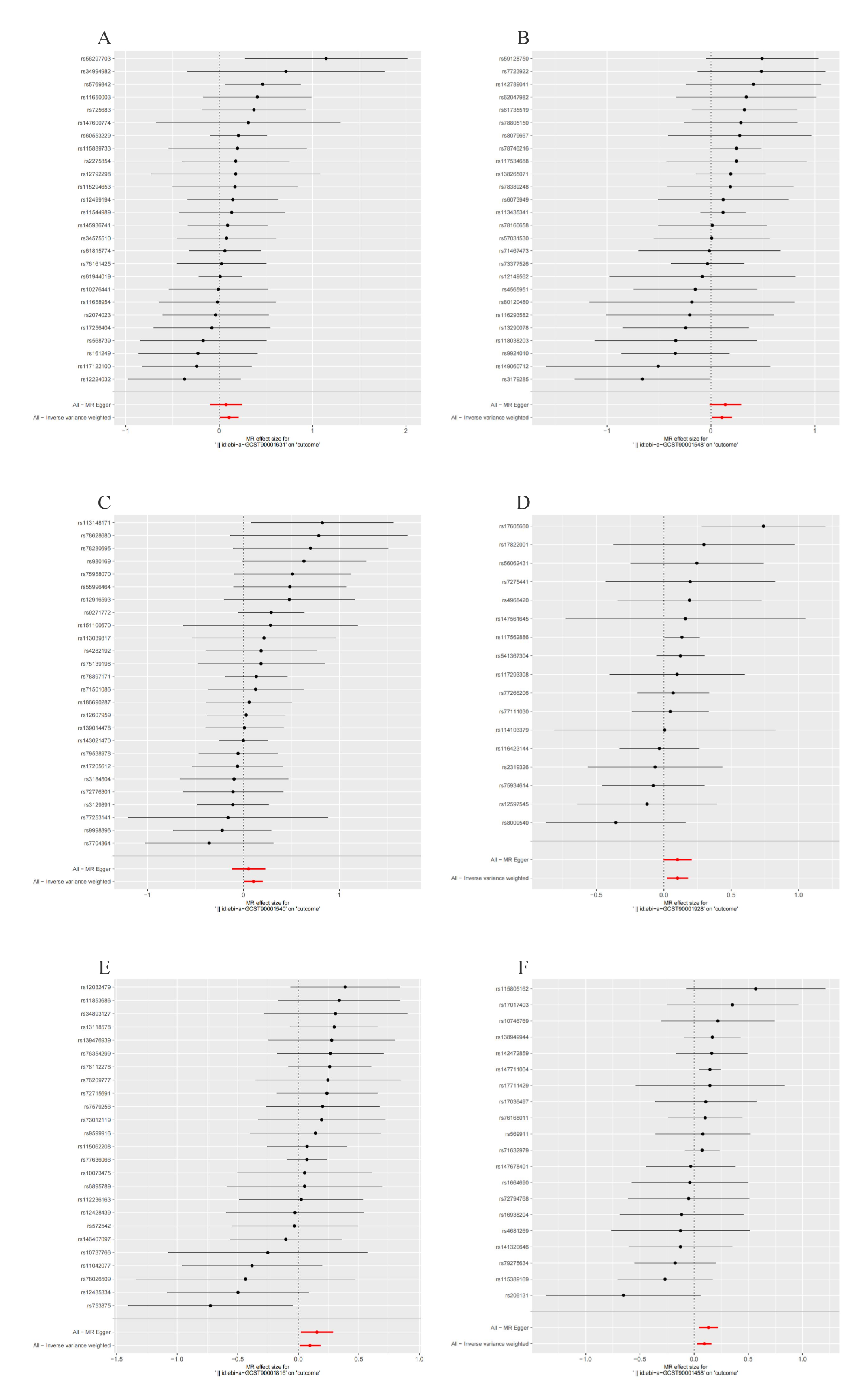

Supplement: Supplementary file 2 [file DataSheet2.zip › Supplement figureS13_S27/Figure_S14.tiff]

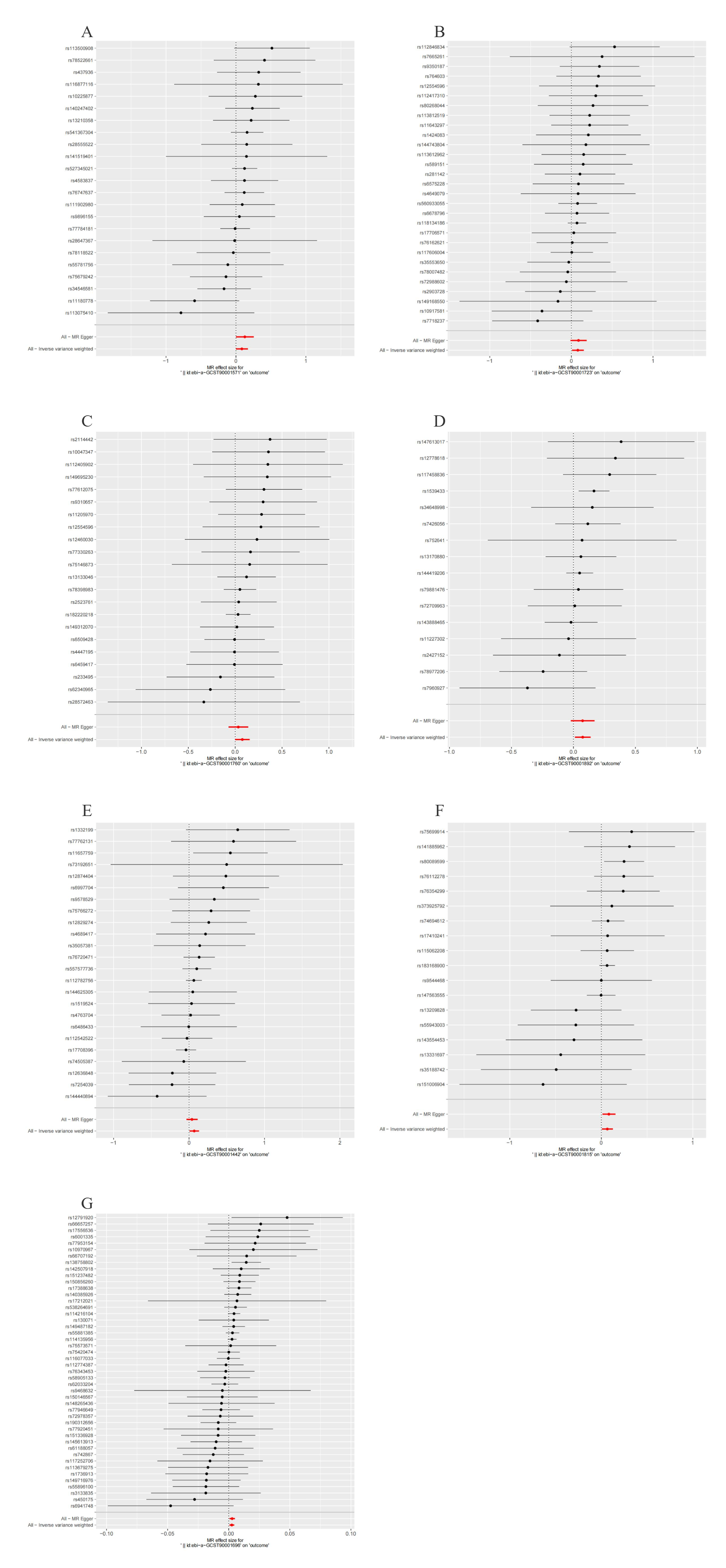

Supplement: Supplementary file 2 [file DataSheet2.zip › Supplement figureS13_S27/Figure_S15.tiff]

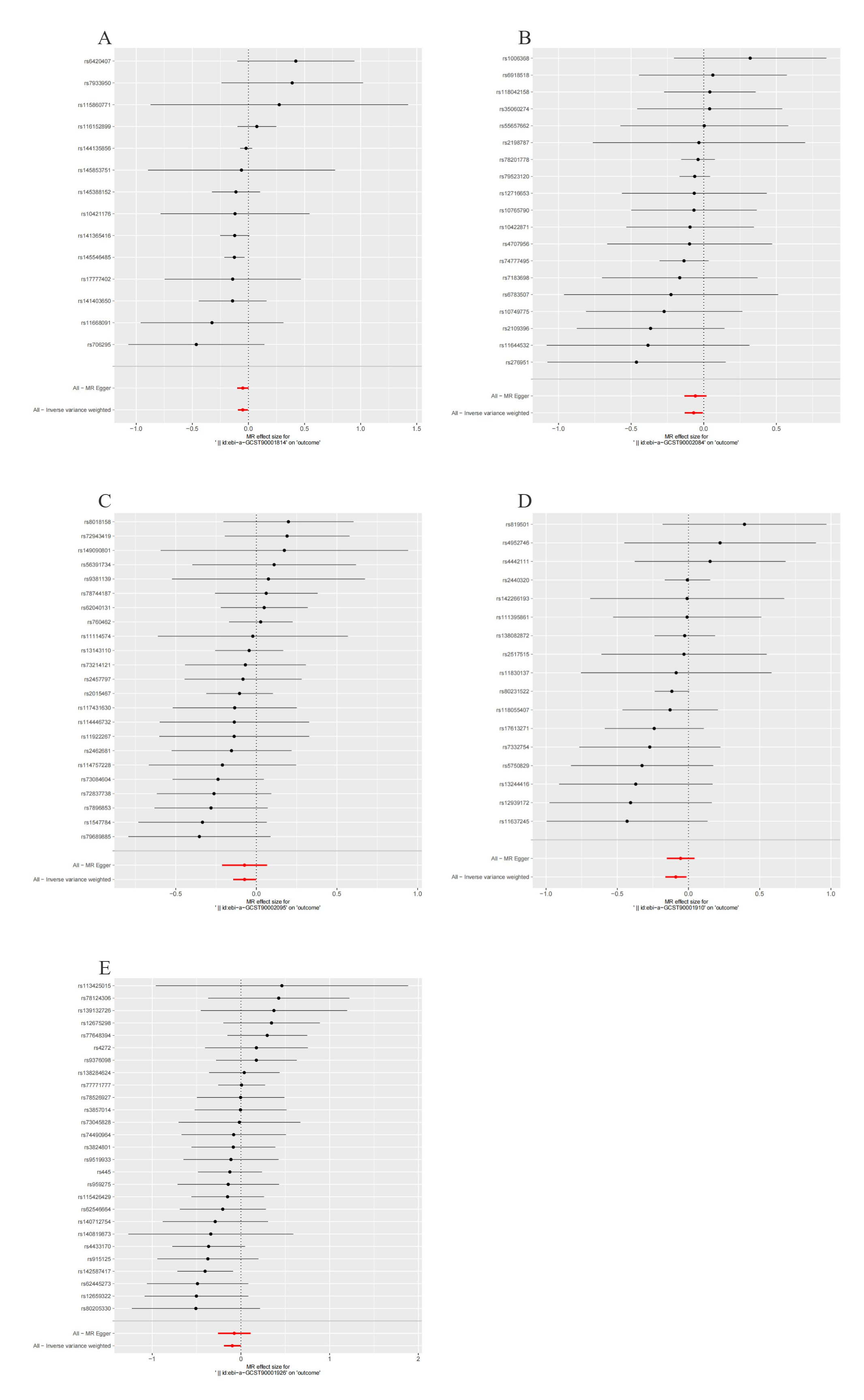

Supplement: Supplementary file 2 [file DataSheet2.zip › Supplement figureS13_S27/Figure_S16.tiff]

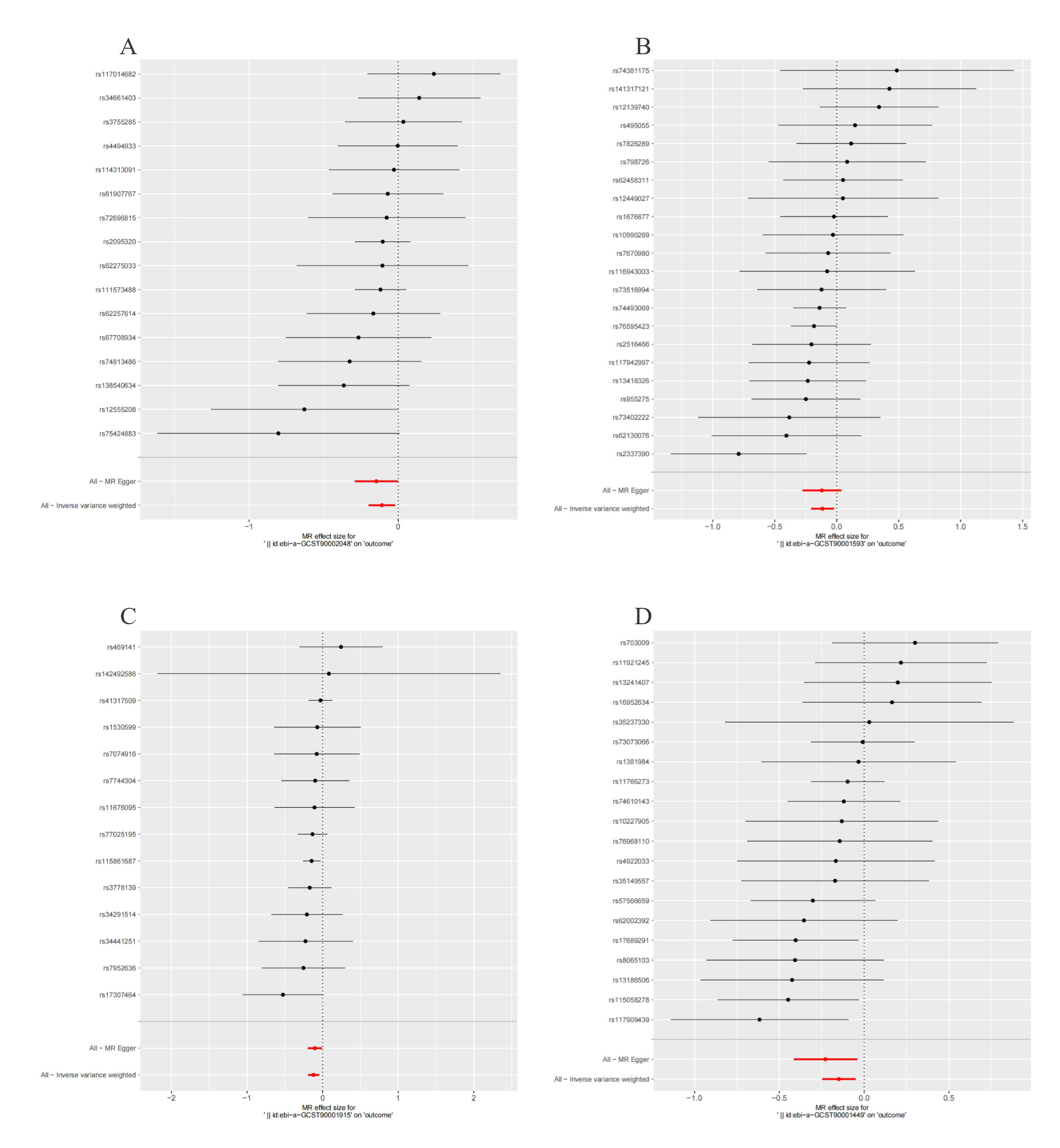

Supplement: Supplementary file 2 [file DataSheet2.zip › Supplement figureS13_S27/Figure_S17.tiff]

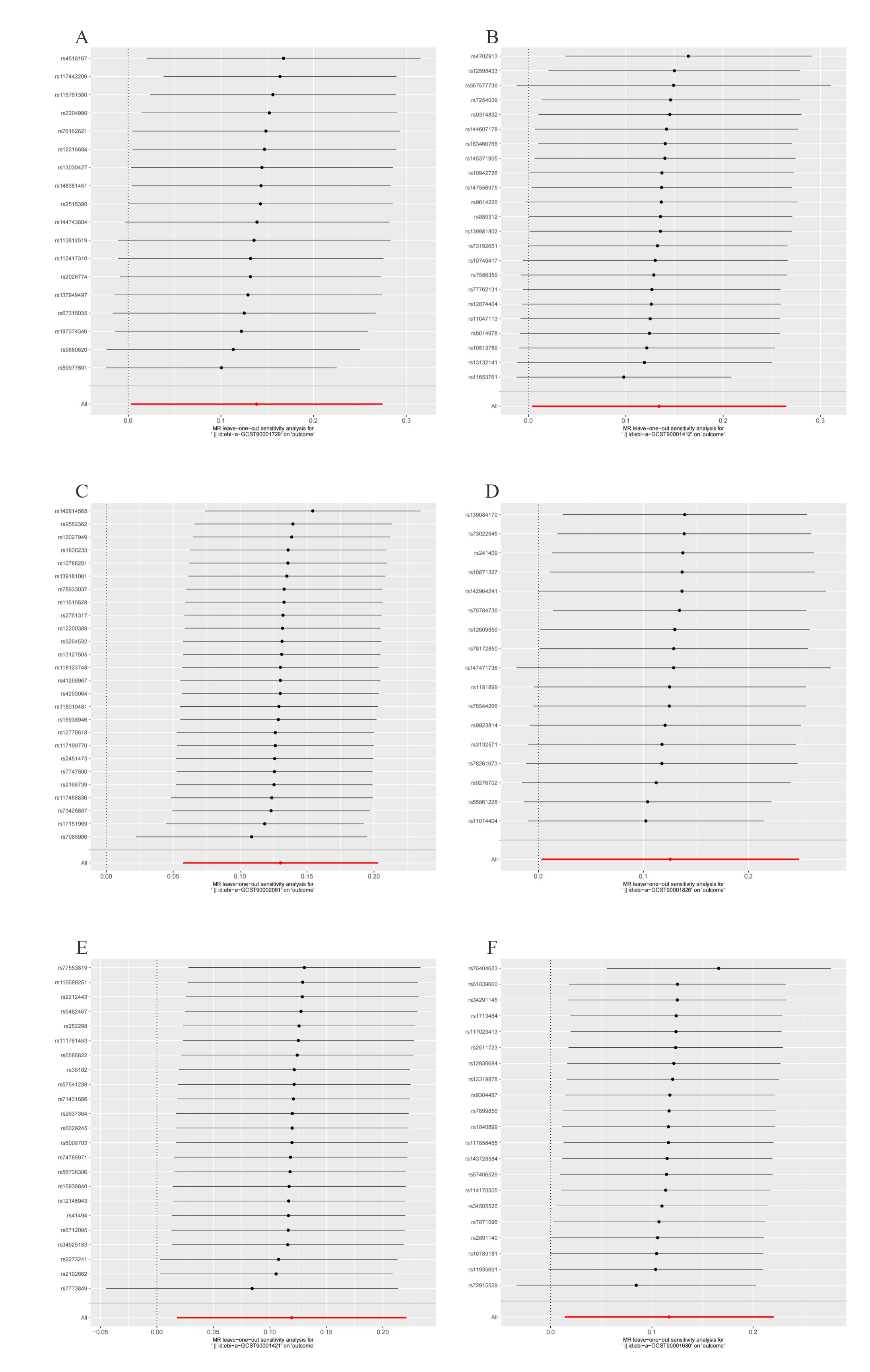

Supplement: Supplementary file 2 [file DataSheet2.zip › Supplement figureS13_S27/Figure_S18.tiff]

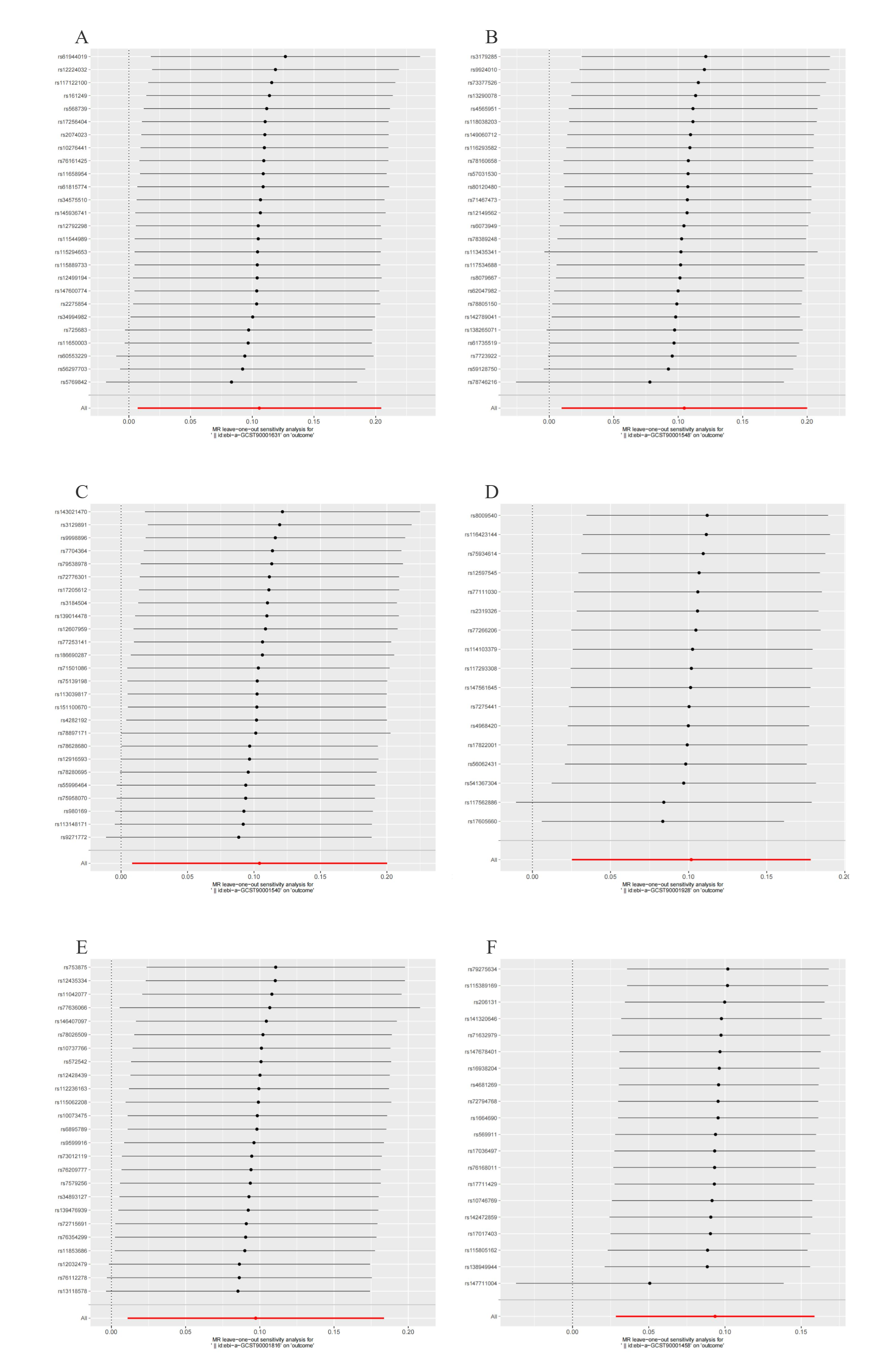

Supplement: Supplementary file 2 [file DataSheet2.zip › Supplement figureS13_S27/Figure_S19.tiff]

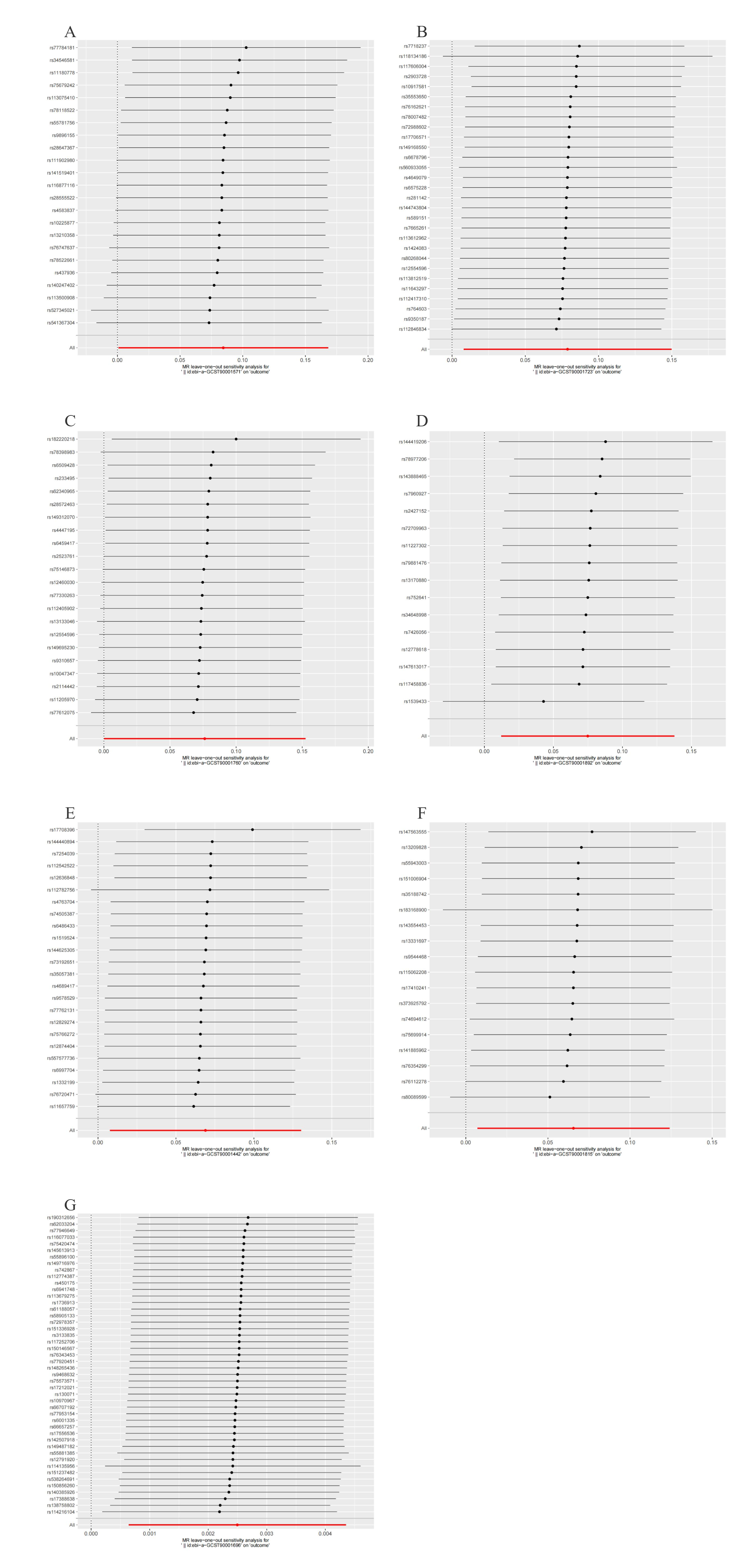

Supplement: Supplementary file 2 [file DataSheet2.zip › Supplement figureS13_S27/Figure_S20.tiff]

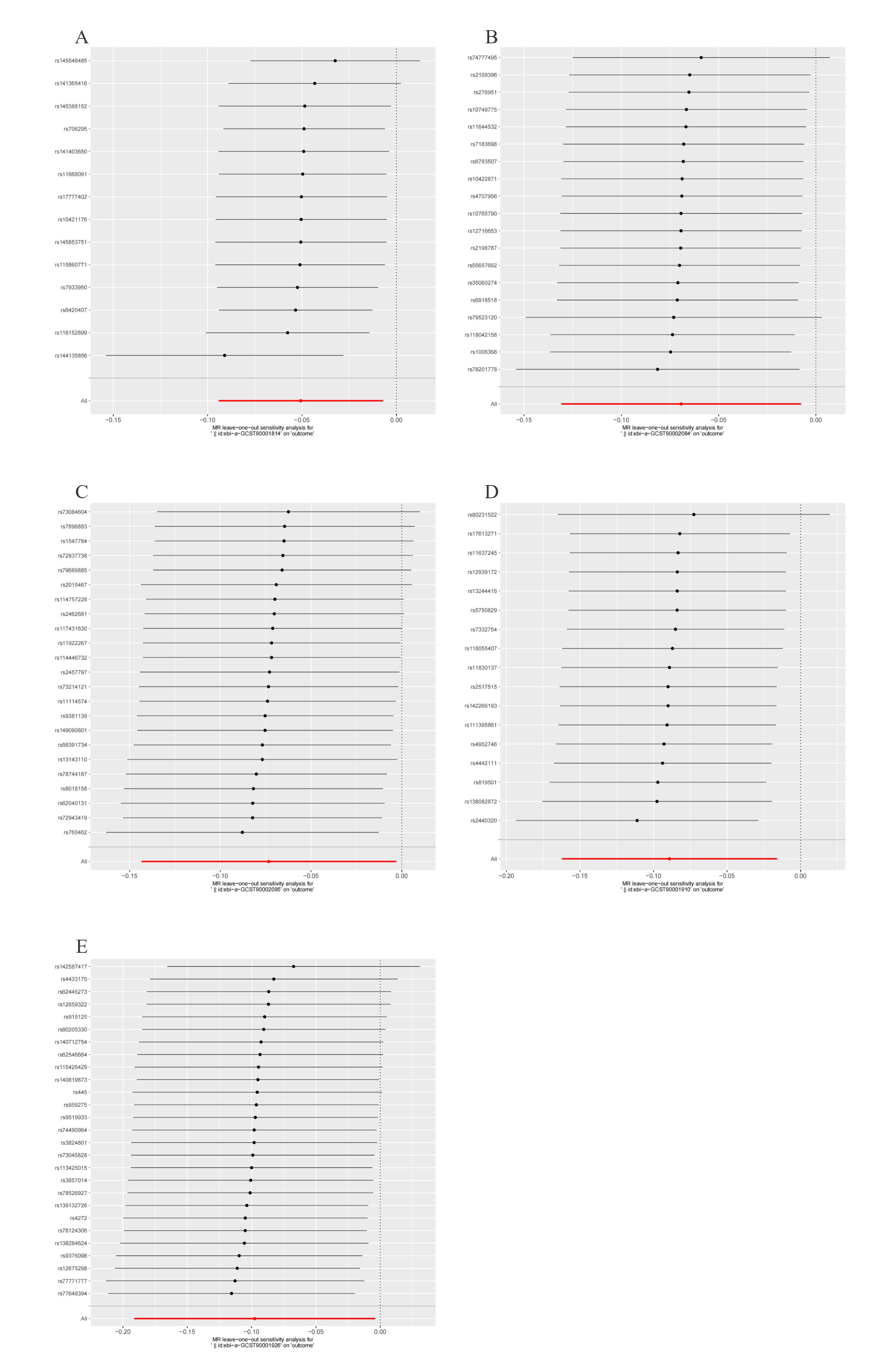

Supplement: Supplementary file 2 [file DataSheet2.zip › Supplement figureS13_S27/Figure_S21.tiff]

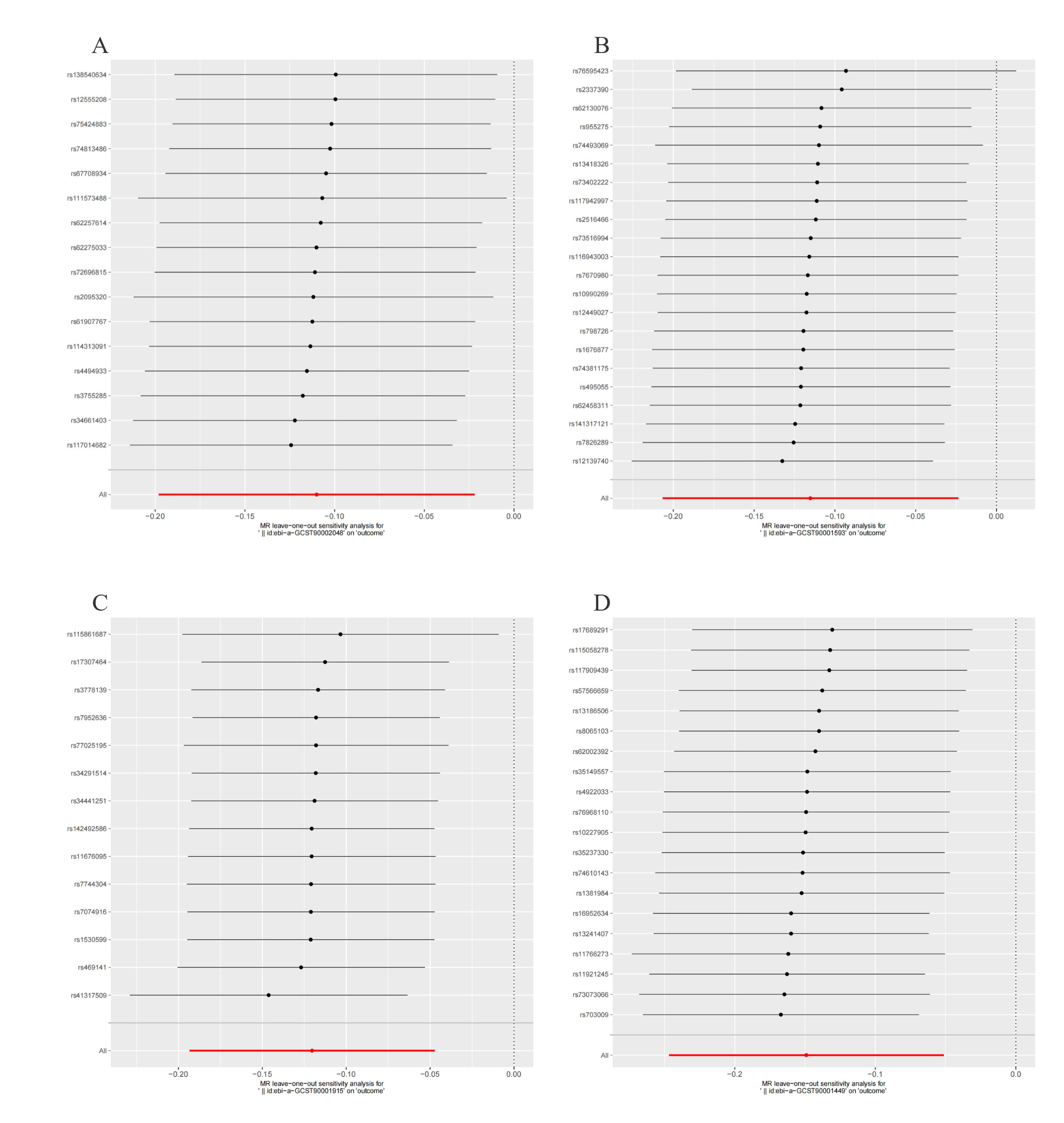

Supplement: Supplementary file 2 [file DataSheet2.zip › Supplement figureS13_S27/Figure_S22.tiff]

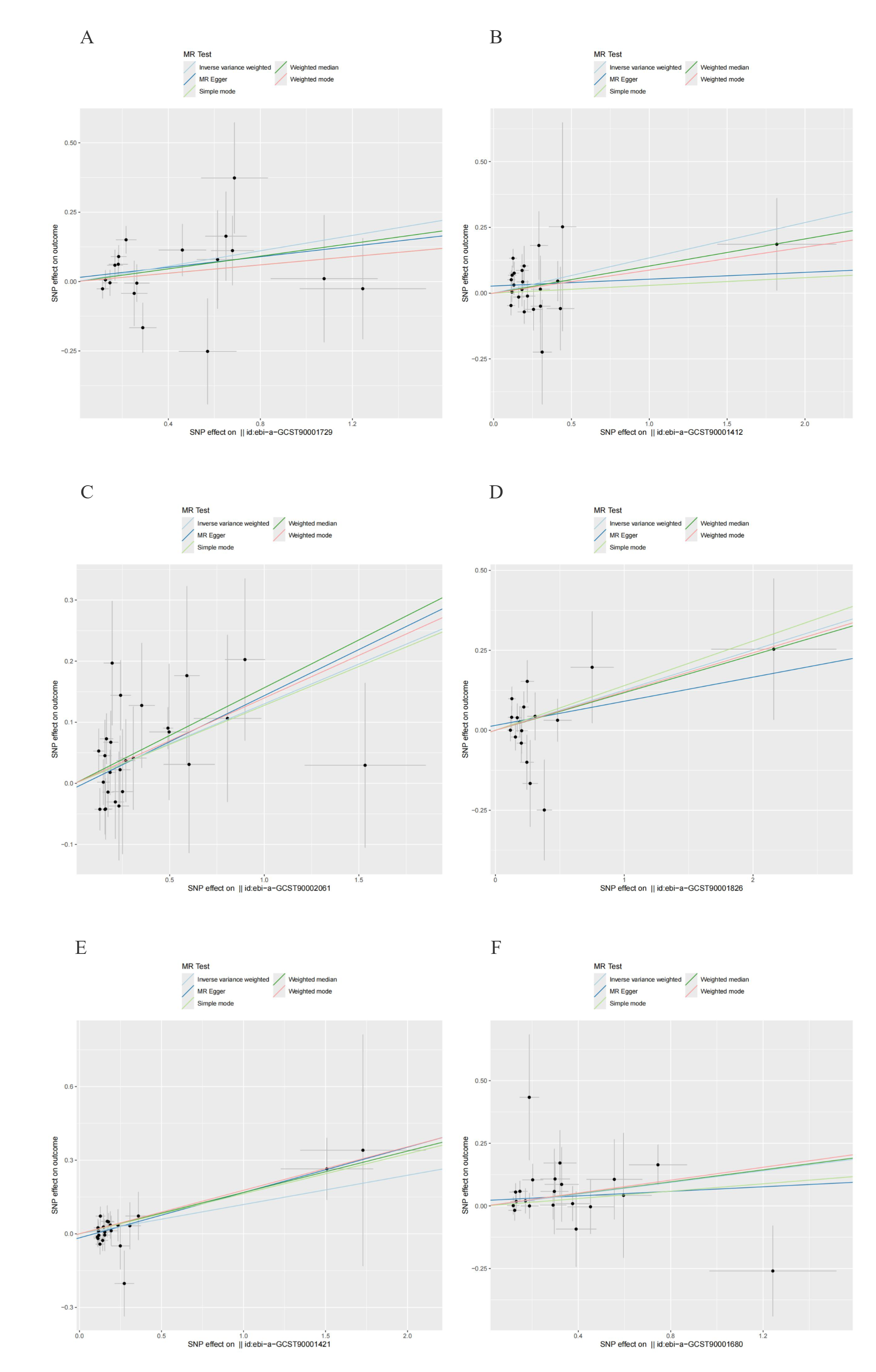

Supplement: Supplementary file 2 [file DataSheet2.zip › Supplement figureS13_S27/Figure_S23.tiff]

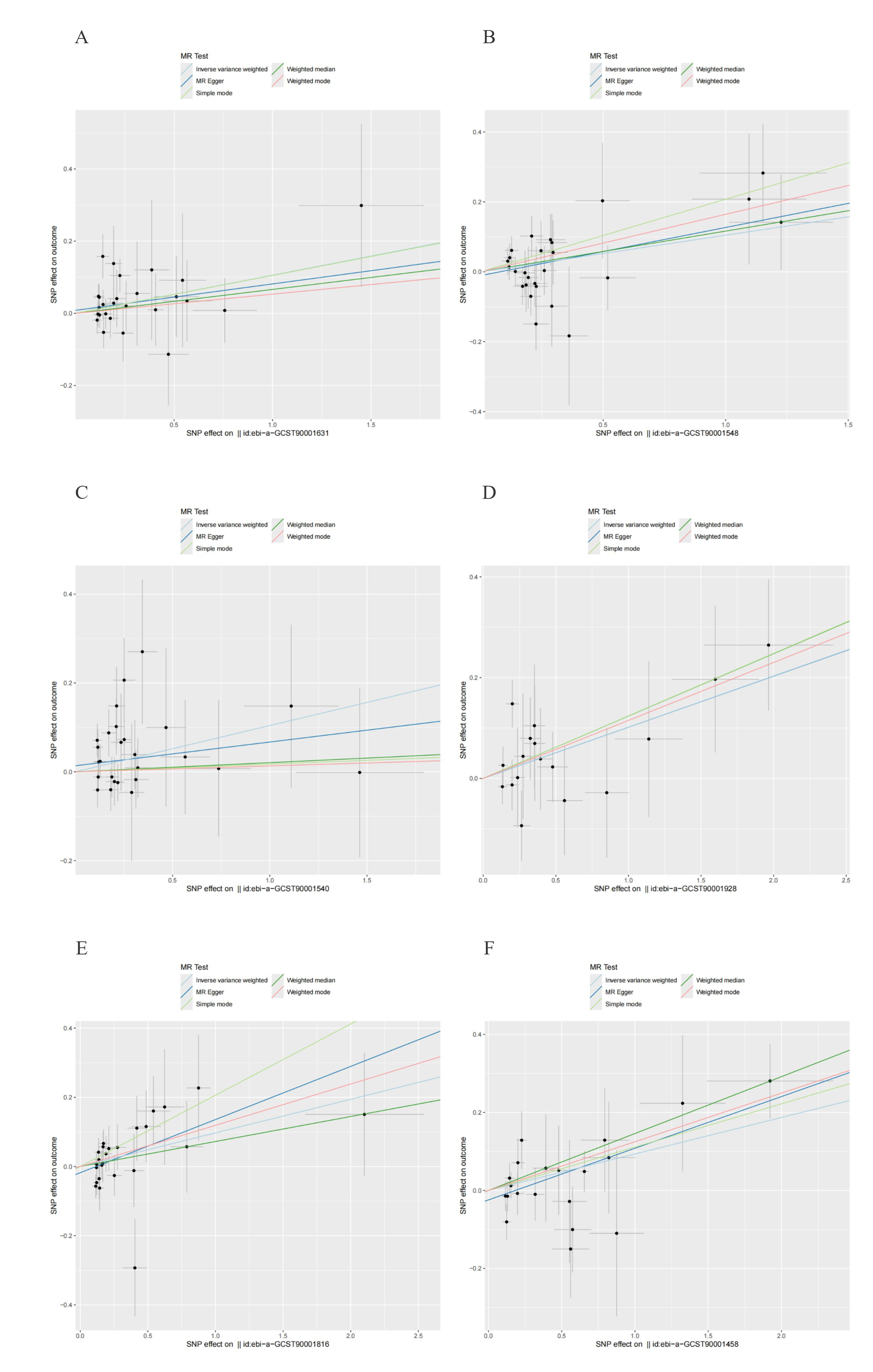

Supplement: Supplementary file 2 [file DataSheet2.zip › Supplement figureS13_S27/Figure_S24.tiff]

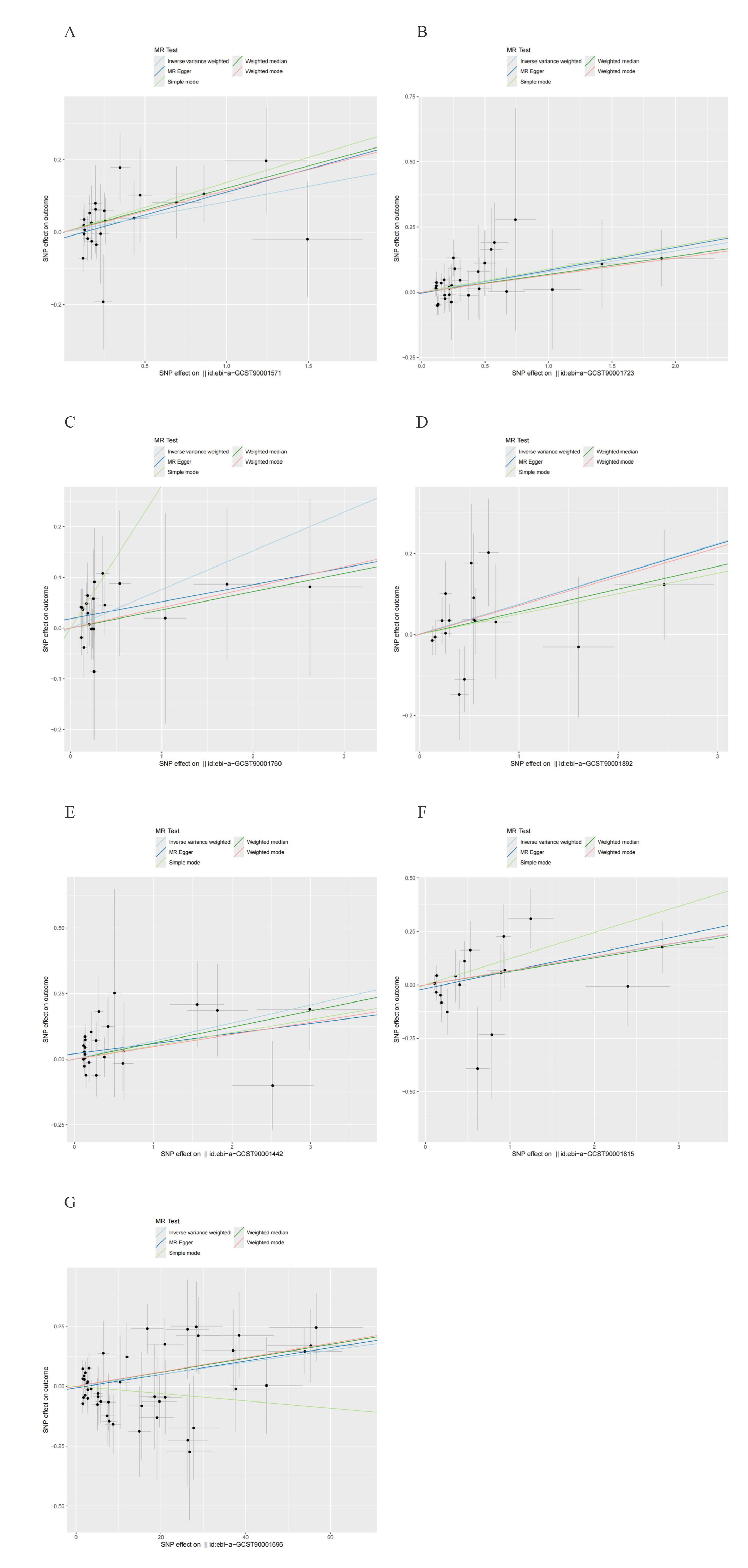

Supplement: Supplementary file 2 [file DataSheet2.zip › Supplement figureS13_S27/Figure_S25.tiff]

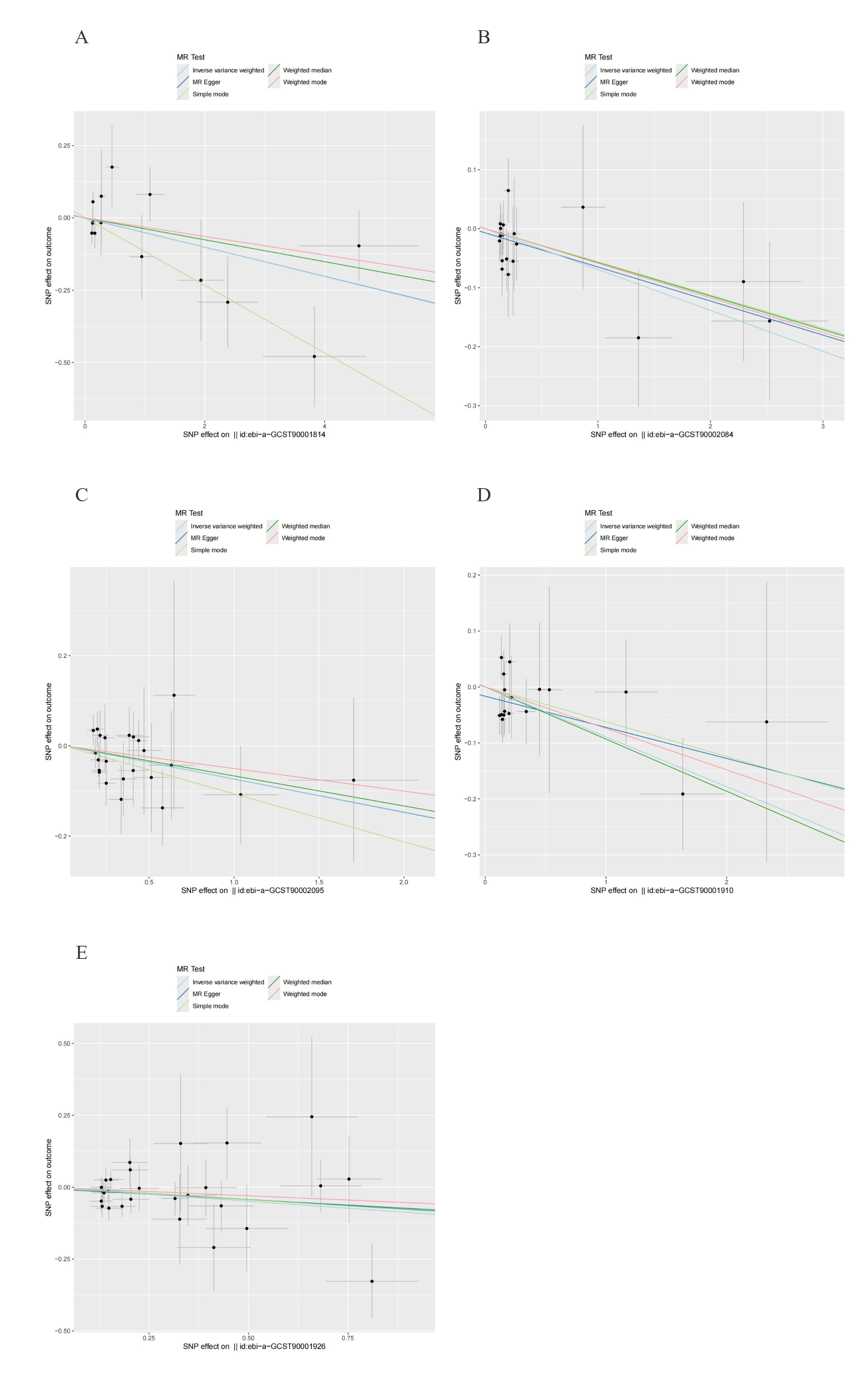

Supplement: Supplementary file 2 [file DataSheet2.zip › Supplement figureS13_S27/Figure_S26.tiff]

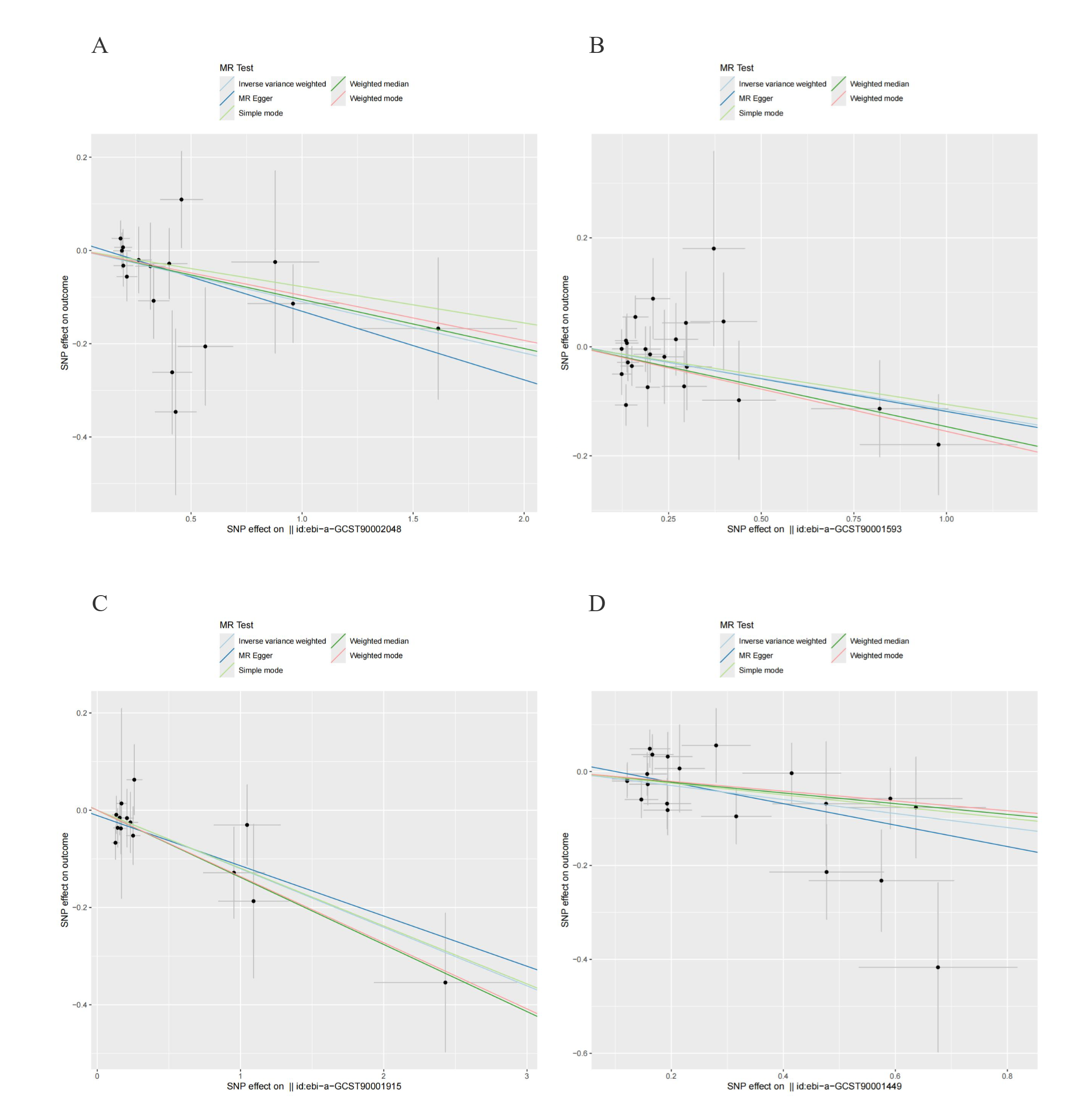

Supplement: Supplementary file 2 [file DataSheet2.zip › Supplement figureS13_S27/Figure_S27.tiff]
