# Supplementary material for: Role of CD38 in mediating the effect of Bacillus on acute pancreatitis: a study of mediated Mendelian randomization
Source: Front Immunol. 2024 Nov 15;15:1452743. doi: 10.3389/fimmu.2024.1452743 (PMC11604618; doi:10.3389/fimmu.2024.1452743)
Supplement: Supplementary file 13 [file Table11.docx]

**Figure S1-4** MR leave-one-out sensitivity analysis for Gut microbiota on AP.

**Figure S1.**

**A：**Analysis for " bacillales" on "AP"

**B：**Analysis for " Planococcaceae" on "AP"

**C:** Analysis for "Hyphomonas" on "AP"

**D:** Analysis for " Pseudomonadales" on "AP"

**E:** Analysis for " Aneurinibacillales" on "AP"

**F:** Analysis for " Clostridium M sp001304855" on "AP"

**Figure S2.**

**A：**Analysis for " Aneurinibacillaceae" on "AP"

**B：**Analysis for " Treponema D" on "AP"

**C:** Analysis for " Enterobacteriaceae" on "AP"

**D:** Analysis for " Bacteroides stercoris" on "AP"

**E:** Analysis for " CAG-81 sp000435795" on "AP"

**Figure S3.**

**A：**Analysis for " Eubacterium R sp000431535" on "AP"

**B：**Analysis for " Acutalibacteraceae sp002438685" on "AP"

**C:** Analysis for " Bifidobacterium angulatum" on "AP"

**D:** Analysis for " Acutalibacteraceae sp002451755" on "AP"

**E:** Analysis for " Faecalicatena glycyrrhizinilyticum" on "AP"

**F:** Analysis for " Faecalicatena sp002161355" on "AP"

**Figure S4.**

**A：**Analysis for " Actinomycetales" on "AP"

**B：**Analysis for " Cyanobacteria" on "AP"

**C:** Analysis for " Johnsonella ignava" on "AP"

**D:** Analysis for " Spirochaetia" on "AP"

**E:** Analysis for " Bacillus C" on "AP"

**Figure S5-8** Scatter plots for the effect of Gut microbiota on AP.

**Figure S5.**

**A：**Scatter plots for " bacillales" on "AP"

**B：**Scatter plots for " Planococcaceae" on "AP"

**C:** Scatter plots for "Hyphomonas" on "AP"

**D:** Scatter plots for " Pseudomonadales" on "AP"

**E:** Scatter plots for " Aneurinibacillales" on "AP"

**F:** Scatter plots for " Clostridium M sp001304855" on "AP"

**Figure S6.**

**A：**Scatter plots for " Aneurinibacillaceae" on "AP"

**B：**Scatter plots for " Treponema D" on "AP"

**C:** Scatter plots for " Enterobacteriaceae" on "AP"

**D:** Scatter plots for " Bacteroides stercoris" on "AP"

**E:** Scatter plots for " CAG-81 sp000435795" on "AP"

**Figure S7.**

**A：**Scatter plots for " Eubacterium R sp000431535" on "AP"

**B：**Scatter plots for " Acutalibacteraceae sp002438685" on "AP"

**C:** Scatter plots for " Bifidobacterium angulatum" on "AP"

**D:** Scatter plots for " Acutalibacteraceae sp002451755" on "AP"

**E:** Scatter plots for " Faecalicatena glycyrrhizinilyticum" on "AP"

**F:** Scatter plots for " Faecalicatena sp002161355" on "AP"

**Figure S8.**

**A：**Scatter plots for " Actinomycetales" on "AP"

**B：**Scatter plots for " Cyanobacteria" on "AP"

**C:** Scatter plots for " Johnsonella ignava" on "AP"

**D:** Scatter plots for " Spirochaetia" on "AP"

**E:** Scatter plots for " Bacillus C" on "AP"

**Figure S9-12** fixed-effect IVW analysis for Gut microbiota on AP.

**Figure S9.**

**A：**Analysis for " bacillales" on "AP"

**B：**Analysis for " Planococcaceae" on "AP"

**C:** Analysis for "Hyphomonas" on "AP"

**D:** Analysis for " Pseudomonadales" on "AP"

**E:** Analysis for " Aneurinibacillales" on "AP"

**F:** Analysis for " Clostridium M sp001304855" on "AP"

**Figure S10.**

**A：**Analysis for " Aneurinibacillaceae" on "AP"

**B：**Analysis for " Treponema D" on "AP"

**C:** Analysis for " Enterobacteriaceae" on "AP"

**D:** Analysis for " Bacteroides stercoris" on "AP"

**E:** Analysis for " CAG-81 sp000435795" on "AP"

**Figure S11.**

**A：**Analysis for " Eubacterium R sp000431535" on "AP"

**B：**Analysis for " Acutalibacteraceae sp002438685" on "AP"

**C:** Analysis for " Bifidobacterium angulatum" on "AP"

**D:** Analysis for " Acutalibacteraceae sp002451755" on "AP"

**E:** Analysis for " Faecalicatena glycyrrhizinilyticum" on "AP"

**F:** Analysis for " Faecalicatena sp002161355" on "AP"

**Figure S12.**

**A：**Analysis for " Actinomycetales" on "AP"

**B：**Analysis for " Cyanobacteria" on "AP"

**C:** Analysis for " Johnsonella ignava" on "AP"

**D:** Analysis for " Spirochaetia" on "AP"

**E:** Analysis for " Bacillus C" on "AP"

**Figure S13-17** MR leave-one-out sensitivity analysis for immune cell traits on AP.

**Figure S13.**

**A：**Analysis for " CD19 on IgD+ CD38br" on "AP"

**B：**Analysis for " IgD+ CD24+ AC" on "AP"

**C:** Analysis for " CD4 on CD39+ CD4+" on "AP"

**D:** Analysis for " IgD on unsw mem" on "AP"

**E:** Analysis for " CD20- AC" on "AP"

**F:** Analysis for " CD25++ CD8br %CD8br" on "AP"

**Figure S14.**

**A：**Analysis for " CD8br NKT %T cell" on "AP"

**B：**Analysis for " CM CD8br AC" on "AP"

**C:** Analysis for " Naive CD4+ AC" on "AP"

**D:** Analysis for " CD127 on CD28+ CD45RA- CD8br" on "AP"

**E:** Analysis for " CD38 on naive-mature B cell" on "AP"

**F:** Analysis for " Myeloid DC AC" on "AP"

**Figure S15.**

**A：**Analysis for " EM DN (CD4-CD8-) %T cell" on "AP"

**B：**Analysis for " CD19 on CD24+ CD27+" on "AP"

**C:** Analysis for " CD20 on unsw mem" on "AP"

**D:** Analysis for " CD28 on CD39+ CD4+" on "AP"

**E:** Analysis for " CD24+ CD27+ %lymphocyte" on "AP"

**F:** Analysis for " CD38 on IgD- CD38dim" on "AP"

**G:** Analysis for " CD45RA- CD28- CD8br %CD8br" on "AP"

**Figure S16.**

**A：**Analysis for " CD38 on IgD- CD38br" on "AP"

**B：**Analysis for " SSC-A on NKT" on "AP"

**C:** Analysis for " CD11b on CD33dim HLA DR-" on "AP"

**D:** Analysis for " CD45 on B cell" on "AP"

**E:** Analysis for " CD127 on granulocyte" on "AP"

**Figure S17.**

**A：**Analysis for " CD45 on CD66b++ myelod cell" on "AP"

**B：**Analysis for " CD8br %T cell" on "AP"

**C:** Analysis for " CD45 on T cell" on "AP"

**D:** Analysis for " CD11c+ monocyte %monocyte" on "AP"

**Figure S18-22** Scatter plots for the effect of immune cell traits on AP.

**Figure S18.**

**A：**Scatter plot for " CD19 on IgD+ CD38br" on "AP"

**B：**Scatter plot for " IgD+ CD24+ AC" on "AP"

**C:** Scatter plot for " CD4 on CD39+ CD4+" on "AP"

**D:** Scatter plot for " IgD on unsw mem" on "AP"

**E:** Scatter plot for " CD20- AC" on "AP"

**F:** Scatter plot for " CD25++ CD8br %CD8br" on "AP"

**Figure S19.**

**A：**Scatter plot for " CD8br NKT %T cell" on "AP"

**B：**Scatter plot for " CM CD8br AC" on "AP"

**C:** Scatter plot for " Naive CD4+ AC" on "AP"

**D:** Scatter plot for " CD127 on CD28+ CD45RA- CD8br" on "AP"

**E:** Scatter plot for " CD38 on naive-mature B cell" on "AP"

**F:** Scatter plot for " Myeloid DC AC" on "AP"

**Figure S20.**

**A：**Scatter plot for " EM DN (CD4-CD8-) %T cell" on "AP"

**B：**Scatter plot for " CD19 on CD24+ CD27+" on "AP"

**C:** Scatter plot for " CD20 on unsw mem" on "AP"

**D:** Scatter plot for " CD28 on CD39+ CD4+" on "AP"

**E:** Scatter plot for " CD24+ CD27+ %lymphocyte" on "AP"

**F:** Scatter plotfor " CD38 on IgD- CD38dim" on "AP"

**G:** Scatter plot for " CD45RA- CD28- CD8br %CD8br" on "AP"

**Figure S21.**

**A：**Scatter plot for " CD38 on IgD- CD38br" on "AP"

**B：**Scatter plot for " SSC-A on NKT" on "AP"

**C:** Scatter plot for " CD11b on CD33dim HLA DR-" on "AP"

**D:** Scatter plot for " CD45 on B cell" on "AP"

**E:** Scatter plot for " CD127 on granulocyte" on "AP"

**Figure S22.**

**A：**Scatter plot for " CD45 on CD66b++ myelod cell" on "AP"

**B：**Scatter plot for " CD8br %T cell" on "AP"

**C:** Scatter plot for " CD45 on T cell" on "AP"

**D:** Scatter plot for " CD11c+ monocyte %monocyte" on "AP"

**Figure S23-27** fixed-effect IVW analysis for immune cell traits on AP.

**Figure S23.**

**A：**Analysis for " CD19 on IgD+ CD38br" on "AP"

**B：**Analysis for " IgD+ CD24+ AC" on "AP"

**C:** Analysis for " CD4 on CD39+ CD4+" on "AP"

**D:** Analysis for " IgD on unsw mem" on "AP"

**E:** Analysis for " CD20- AC" on "AP"

**F:** Analysis for " CD25++ CD8br %CD8br" on "AP"

**Figure S24.**

**A：**Analysis for " CD8br NKT %T cell" on "AP"

**B：**Analysis for " CM CD8br AC" on "AP"

**C:** Analysis for " Naive CD4+ AC" on "AP"

**D:** Analysis for " CD127 on CD28+ CD45RA- CD8br" on "AP"

**E:** Analysis for " CD38 on naive-mature B cell" on "AP"

**F:** Analysis for " Myeloid DC AC" on "AP"

**Figure S25.**

**A：**Analysis for " EM DN (CD4-CD8-) %T cell" on "AP"

**B：**Analysis for " CD19 on CD24+ CD27+" on "AP"

**C:** Analysis for " CD20 on unsw mem" on "AP"

**D:** Analysis for " CD28 on CD39+ CD4+" on "AP"

**E:** Analysis for " CD24+ CD27+ %lymphocyte" on "AP"

**F:** Analysis for " CD38 on IgD- CD38dim" on "AP"

**G:** Analysis for " CD45RA- CD28- CD8br %CD8br" on "AP"

**Figure S26.**

**A：**Analysis for " CD38 on IgD- CD38br" on "AP"

**B：**Analysis for " SSC-A on NKT" on "AP"

**C:** Analysis for " CD11b on CD33dim HLA DR-" on "AP"

**D:** Analysis for " CD45 on B cell" on "AP"

**E:** Analysis for " CD127 on granulocyte" on "AP"

**Figure S27.**

**A：**Analysis for " CD45 on CD66b++ myelod cell" on "AP"

**B：**Analysis for " CD8br %T cell" on "AP"

**C:** Analysis for " CD45 on T cell" on "AP"

**D:** Analysis for " CD11c+ monocyte %monocyte" on "AP"
